# Single-Phase Ternary Compounds with a Disordered Lattice and Liquid Metal Phase for High-Performance Li-Ion Battery Anodes

**DOI:** 10.1007/s40820-023-01026-4

**Published:** 2023-03-10

**Authors:** Yanhong Li, Lei Zhang, Hung-Yu Yen, Yucun Zhou, Gun Jang, Songliu Yuan, Jeng-Han Wang, Peixun Xiong, Meilin Liu, Ho Seok Park, Wenwu Li

**Affiliations:** 1 School of Chemical Engineering, Sungkyunkwan University, 2066, Seobu-ro, Jangan-gu, Suwon-Si, Gyeonggi-Do 440-746 Korea; 2 School of Physics, Huazhong University of Science and Technology, Wuhan, 430074 People’s Republic of China; 3 School of Materials Science and Engineering, Georgia Institute of Technology, Atlanta, GA 30332 USA; 4 Department of Chemistry, National Taiwan Normal University, Taipei, 11677 Taiwan, People’s Republic of China; 5 Samsung Advanced Institute for Health Sciences and Technology (SAIHST), Sungkyunkwan University, 2066 Seoburo, Jangan-gu, Suwon, 440-746 Korea; 6 SKKU Advanced Institute of Nano Technology (SAINT), Sungkyunkwan University , 2066 Seoburo, Jangan-gu, Suwon, 440-746 Korea; 7 SKKU Institute of Energy Science and Technology (SIEST), Sungkyunkwan University , 2066 Seoburo, Jangan-gu, Suwon, 440-746 Korea

**Keywords:** Multinary compounds, Liquid metal, GaSiP_2_, Disordered lattice, Li-ion batteries

## Abstract

Si is considered as the promising anode materials for lithium-ion batteries (LIBs) owing to their high capacities of 4200 mAh g^−1^ and natural abundancy. However, severe electrode pulverization and poor electronic and Li-ionic conductivities hinder their practical applications. To resolve the afore-mentioned problems, we first demonstrate a cation-mixed disordered lattice and unique Li storage mechanism of single-phase ternary GaSiP_2_ compound, where the liquid metallic Ga and highly reactive P are incorporated into Si through a ball milling method. As confirmed by experimental and theoretical analyses, the introduced Ga and P enables to achieve the stronger resistance against volume variation and metallic conductivity, respectively, while the cation-mixed lattice provides the faster Li-ionic diffusion capability than those of the parent GaP and Si phases. The resulting GaSiP_2_ electrodes delivered the high specific capacity of 1615 mAh g^−1^ and high initial Coulombic efficiency of 91%, while the graphite-modified GaSiP_2_ (GaSiP_2_@C) achieved 83% of capacity retention after 900 cycles and high-rate capacity of 800 at 10,000 mA g^−1^. Furthermore, the LiNi_0.8_Co_0.1_Mn_0.1_O_2_//GaSiP_2_@C full cells achieved the high specific capacity of 1049 mAh g^−1^ after 100 cycles, paving a way for the rational design of high-performance LIB anode materials.

**Supplementary Information:**

The online version contains supplementary material available at 10.1007/s40820-023-01026-4.

## Highlights

- Single-phase ternary GaSiP_2_with a disordered lattice and liquid metal was synthesized.
- GaSiP_2_ electrode achieved superior Li-storage performances in both half and full cells.
- Unique self-healing Li-storage mechanism of GaSiP_2_ was analyzed.

**Supplementary Information:**

The online version contains supplementary material available at 10.1007/s40820-023-01026-4.

## Graphical abstract

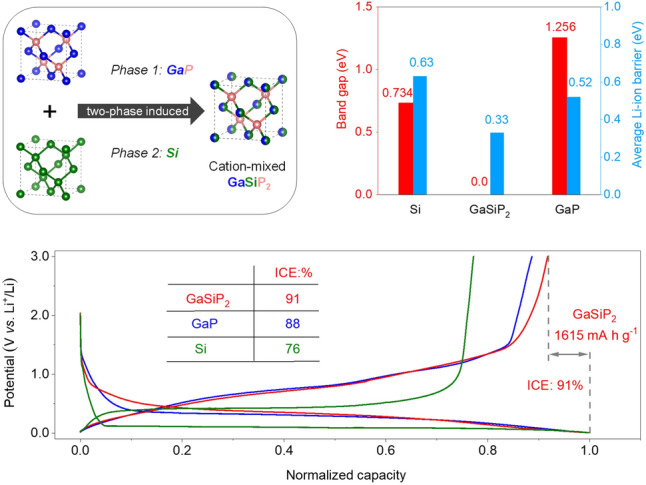

## Introduction

Developing advanced anode materials with the large capacity, high-rate capability, and appropriate potential is crucial for replacing the current commercialized graphite that endures the limited capacity, sluggish ion diffusion, and potential dendrite growth by the low working potential [1–3]. Si and P are regarded as the promising anode materials for LIBs owing to their high capacities (4200 and 2596 mAh g^−1^ corresponding to Li_22_Si_4_ and Li_3_P, respectively), and abundant reserves [4–12]. However, severe electrode pulverization and poor electronic and Li-ionic conductivities hinder their practical applications [13–15]. Compared with Si, Si–P-based compounds show faster reaction kinetics and better safety due to the inherent metallic phase of cubic Si_3_P, SiP and SiP_2_, *in situ* formed Li-ionic conductors of Li_3_P and others, and higher operating potentials [16, 17]. However, the severe electrode pulverization still remains a critical challenge. The liquid metals with self-healing property have been exploited to improve the cycling stability of rechargeable batteries owing to the deformation of the liquid phase, the surface tension, and the fluidity [18–21]. For instance, the cracks caused upon the repeated Li-ion insertion and extraction could be healed by means of the phase change of the Ga-based compounds originating from the low melting temperature of 29.76 °C, which is within the operating temperature range of the working batteries [22–26]. Importantly, the Li-ion diffusion coefficient in liquid Ga is about 1.4 × 10^–9^ cm^2^ s^−1^, which is two orders of magnitude higher than that in Si [27, 28]. Therefore, the introduction of liquid metal Ga into Si–P-based compounds is postulated to resolve the electrode pulverization and to improve electronic and Li-ionic conductivities.

Recently, the single-phase multiple compounds with the unique physical and chemical properties have received significant attention owing to the tunable composition and structure. For example, the ternary single-phase CoPSe compounds have the advantages in terms of the faster electronic and ionic transport capability and the reduced mechanical stress, thus leading to higher rate and cycling performances than those of the binary single-phase CoSe_2_ compounds [29]. The introduced phosphorus also enhanced the reactivity and the reversible capacity with the lowered working potentials [30]. Moreover, the ternary single-phase Bi_2_O_2_Se compounds achieved a large number of anion vacancies endowed by the mixed anions, which resulted in achieving better electrochemical performances than those of the corresponding binary Bi_2_O_3_ and Bi_2_Se_3_ counterparts [31].

The structure and composition of multinary compounds determine their electrochemical performances. When cations are enriched in bi-metallic compounds, their electrochemical performances such as the reversible capacity, high rate capability, and long-term cyclability could be greatly improved [32]. Very recently, the cation-mixed arrangement of Li_1.211_Mo_0.467_Cr_0.3_O_2_ with a rock-salt-type structure has shown the facilitated Li-ion diffusion on a basis of the percolation theory [33–36]. Moreover, the cation-mixed Li_3_V_2_O_5_ compounds achieved relatively low Li-ion diffusion barrier energies, thus delivering a faster charging capability than those of graphite and lithium titanate anodes [37]. However, the reversible capacity of the Li_3_V_2_O_5_ compound based on the intercalation reactions is relatively lower than those of conversion-type or alloy-type compounds. Therefore, high-capacity cation-mixed anode materials should be developed while resolving the issues of electrode pulverization and poor electronic and Li-ionic conductivities.

Herein, we report a cation-mixed disordered lattice of single-phase ternary GaSiP_2_ compound, where the liquid metallic Ga and highly reactive P are incorporated into Si through a ball milling method, resolving the issues of severe electrode pulverization, poor electronic conductivity, and sluggish Li-ionic diffusion of Si anodes. The introduced Ga and P enables to achieve the stronger resistance against volume variation and metallic conductivity, respectively, while the cation-mixed lattice provides the faster Li-ionic diffusion capability than those of the parent GaP and Si phases. Thus, the single-phase cation-mixed GaSiP_2_ compound delivered a reversible capacity of 1615 mAh g^−1^, a working potential of 0.45 V, and an initial Coulombic efficiency (ICE) of 91% based on an intercalation-conversion Li-storage mechanism as validated by *in situ* X-ray diffraction (XRD) and *ex situ* XRD, Raman, and X-ray photoelectron spectroscopy (XPS) analyses.

## Experimental Section

### Materials Preparations

Cation-mixed GaSiP_2_ sample and the ball milled intermediates of GaP + Si + P@*xh*, where *x* is the milling time, were prepared by ball milling raw materials of GaP, Si, and amorphous red P, at the grinding speed of 1000 rpm. The mass ratio of the grinding balls to raw materials is about 20:1. The ball milling was proceeded under the protection of argon. The GaSiP_2_@C composite was synthesized, further performing ball milling at a weight ratio (GaSiP_2_:graphite) of 6:3 for 3 h.

### Materials Characterizations

The as-prepared samples were characterized by XRD (Bruker D80), Raman spectrometer (WTEC ALPHA300 with the 532 nm excitation laser), XPS (Thermo Fisher Escalab 250 with monochromatic 150 W Al Ka radiation), field-emission scanning election microscope (FESEM, FEI Quanta650), high-resolution field-emission transmission electron microscope (HRTEM, FEI, Talos F200X), inductively coupled plasma optical emission spectrometer (ICP-OES, Agilent 720), thermo-gravimetry-differential scanning calorimetry instrument (SDT2960, USA).

### Electrochemical Measurements

The active material of GaSiP_2_ was mixed with acetylene black and lithium polyacrylate (Li-PAA, obtained by acid–base neutralization reaction between polyacrylic acid and lithium hydroxide) at the mass ratio of 7:2:1. After mixing, the slurry was coated onto the copper foil and dried in a vacuum oven for 12 h at 70 °C. The GaSiP_2_@C composite was milled with Li-PAA at the weight ratio of 9:1. The loading materials is ~ 1.2 ± 0.3 mg cm^−2^. The CR2032 coin cells were assembled using 1 M LiPF_6_ in ethylene carbonate (EC), diethyl carbonate (DEC), and dimethyl carbonate (DMC) (EC: DEC: DMC, 1:1:1 by volume) as the electrolyte and Li foil as the counter and reference electrode in the glovebox filled with argon (below 0.01 ppm of H_2_O and O_2_). As for full cells, the total capacity of the anode is ~ 1.2 times higher than cathode. The galvanostatic charging and discharging (GCD) and galvanostatic intermittent titration technique (GITT) tests were conducted by using a LAND testing system. The cyclic voltammetry (CV) curves were collected using the electrochemical workstation (Autolab, Pgstat 302N). *In situ* half-cell were assembled for *in situ* XRD characterizations equipped with Be window and fiberglass separator (LIB-LHTXRD-LN, Beijing Scistar Technology Co., Ltd.) to investigate the crystal structure evolution of the GaSiP_2_ compound electrode.

### Theoretical Calculations

Theoretical calculations were operated by applying Vienna Abinitio Simulation Package (VASP) according to first principles calculations [38]. The exchange correlation function was made using generalized gradient approximation [39], which is proposed by Perdew, Burke, and Ernzerhof. For enough energy calculations, the 4 × 4 × 4 Monkhorst–Pack reciprocal grid along with 400 eV energy cutoff was utilized. The core-electrons were maintained frozen in the way of projector augmented wave function, with the valence electron conformation for Ga 3*d*^10^4*s*^2^*p*^1^, Si 3*s*^2^*p*^2^, and P 3*s*^2^*p*^3^. Gaussian smearing with a smearing width of 0.05 eV was served for speeding up the computation of the electronic energy near the Fermi level.

## Results and Discussion

### Synthesis and Characterization of Ternary Cation-mixed GaSiP_2_ Compounds

**Fig. 1:**
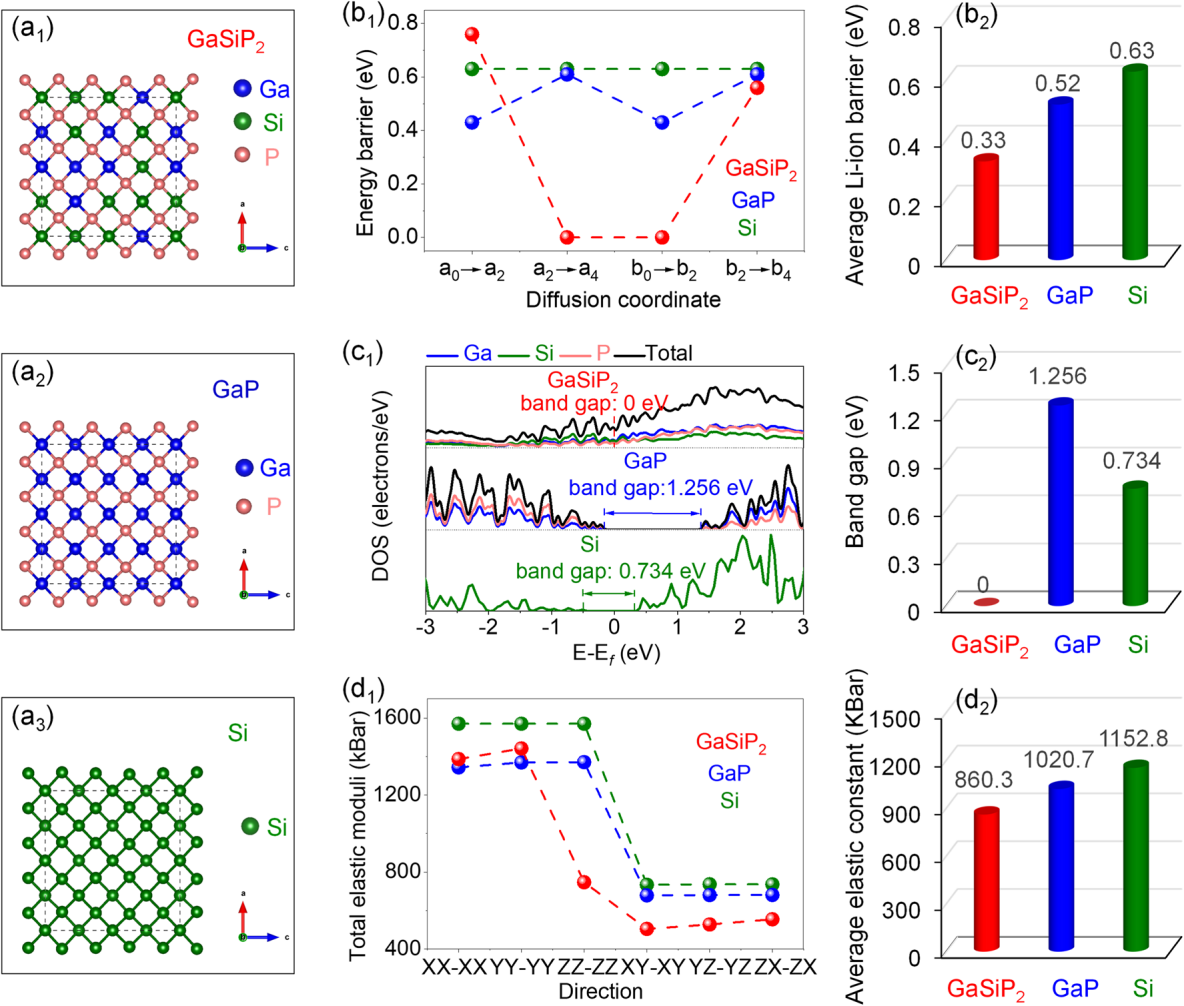
Physicochemical properties resolved by first-principles calculations of the cation-mixed GaSiP_2_ compound, GaP and Si: **a** 64-atom supercell; **b** Li-ion diffusion barrier energies; **c** DOS; **d** Elastic moduli

Based on percolation theory, the diffusion of Li-ions within the ternary cation-mixed GaSiP_2_ compound with a mixed-lattice structure is much faster than that with an ordered lattice [33]. In this study, the density functional theory based on first-principles calculations was carried out to analyze the supercell models of the cation mixed GaSiP_2_, GaP, and Si as shown in Fig. 1a. The cation mixed GaSiP_2_ compound model is constructed using the special quasi-random structure (SQS) method [40, 41]. Considering the major contribution of Li-ionic transport to the Li-storage properties of anode material, we have simulated the comparative Li-ion diffusion paths and energy barriers of the cation-mixed GaSiP_2_ compound, GaP, and Si, as described in Figs. 1b_1_–b_2_, S1, and Table S1. The diffusion barrier energy of Li-ions within the cation-mixed GaSiP_2_ compound is 0.33 eV, much lower than those of the parent phases of both GaP (0.52 eV) and Si (0.63 eV), indicating a faster Li-ionic transport capability of cation-mixed lattice [42]. Along with the Li-ionic transport ability, the electronic conductivity is another vital factor to determine the Li-storage performances because the electron transfer is always accompanied with the Li-ion diffusion through the faradaic reactions. As shown in Fig. 1c_1_–c_2_, the calculated total density of states (TDOS) value of the cation mixed GaSiP_2_ compound at the Fermi level is not equal to zero, suggesting its closed band gap for the metallic conductivity. On the other hand, its parent GaP and Si phases show the semiconductor characteristics, where band gaps are 1.256 and 0.734 eV, respectively. These calculated electronic structures demonstrate that the cation mixed GaSiP_2_ compound has superior electronic conductivity to the GaP and Si counterparts. Finally, resistance to volume expansion is also an important factor to dramatically influence the cycling stability of anodes. It notes that the modulus of elasticity in tension is a mechanical property that measures the tensile stiffness of a solid material when the force is applied lengthwise. As shown in Fig. 1d_1_–d_2_ and Table S2, the cation-mixed GaSiP_2_ compound has smaller elastic constants, which means it is less stiff and much softer than GaP and Si, thus being conducive to the Li-ion diffusion and more tolerant to the volume variation upon cycling [43]. Theoretically, the cation mixed GaSiP_2_ compound achieves faster charge transfer kinetics and better tolerance against volume change than GaP and Si, thus promising to be a high-performance anode material.

**Fig. 2:**
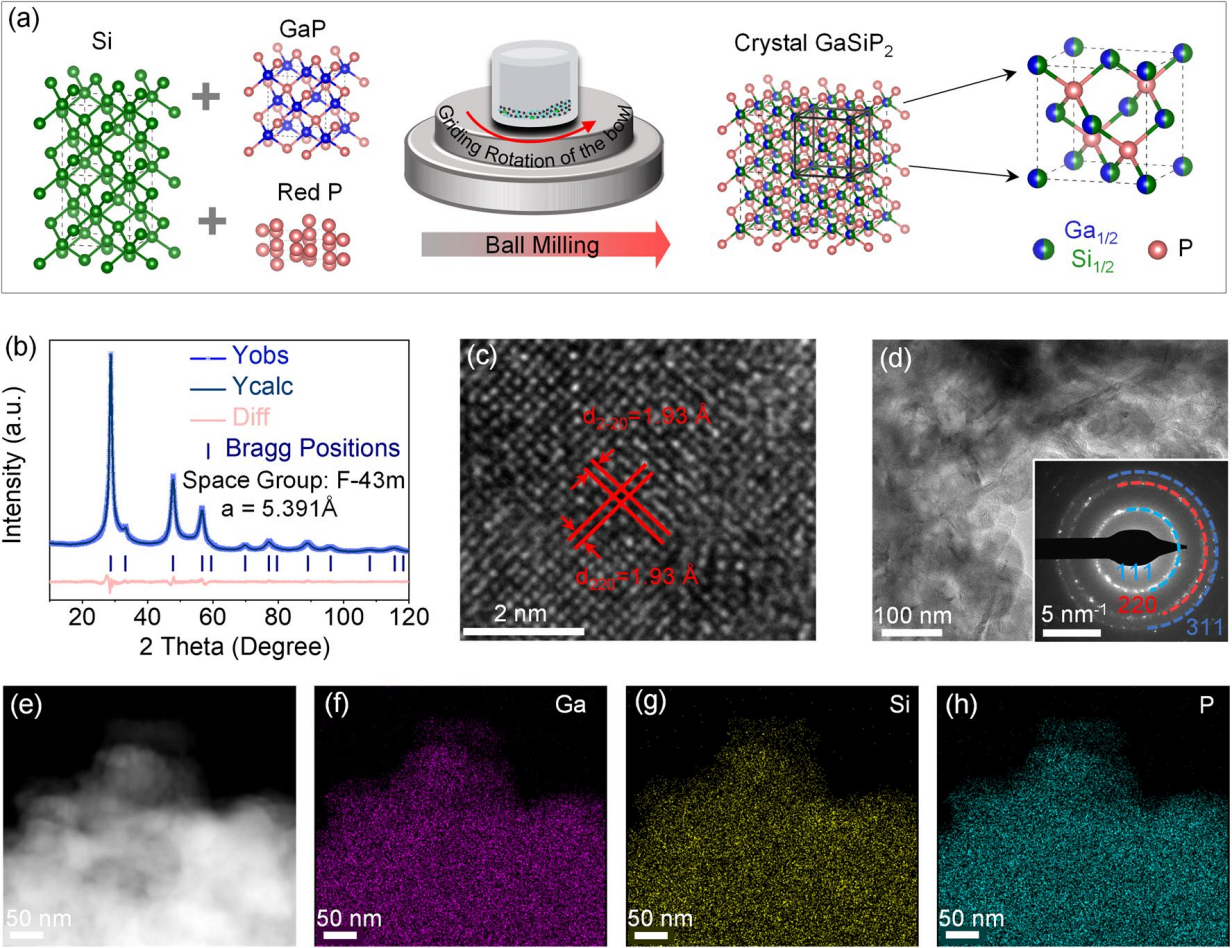
**a** Schematic of the synthesis diagram; **b** XRD refinement; **c**–**d** HRTEM images with SAED pattern (inset of **d**); **e**–**h** Element mapping images of the cation-mixed GaSiP_2_ compound

As shown in Fig. 2a, the ternary cation mixed GaSiP_2_ compound was synthesized via a simple mechanical ball milling method, using the cheap raw materials of GaP, Si, and amorphous red P as precursor powders. The XRD patterns of the ball milled intermediates of GaP + Si + P@*x*h samples are like those of GaP and Si counterparts due to the very similar crystallographic characteristics of the latter. The shift of the strongest peaks is observed under the exposure of different ball milling durations, indicating the formation of a new phase with the similar crystalline structure of GaP and Si (Fig. S2). As presented in Fig. 2b, crystallography refinement was performed to confirm a cation-mixed cubic ZnS structure of the as-prepared GaSiP_2_ compound (GaP + Si + P@8 h) [44, 45], where Ga and Si competitively occupy the cationic sites and P is located at the anionic site (see the detailed refinement information in Tables S3 and S4). As shown in Fig. 2c, HRTEM measurement was performed to detect more accurate local microstructure. The cross-lattice fringe spacing is measured as 0.193 nm, corresponding to (220) and (2–20) planes. The SAED pattern as marked in Fig. 2d presents halo-like multiple rings, indicating the polycrystalline structure as further confirmed by the XRD results. As shown in the elemental mapping images in Fig. 2e–h, the Ga, Si, and P are well distributed, conforming to the high-angle annular dark field scanning TEM (HAADF-STEM) framework. No segregation of any component within the cation mixed GaSiP_2_ compound is indicative of the formation of a new phase after the ball milling process.

**Fig. 3:**
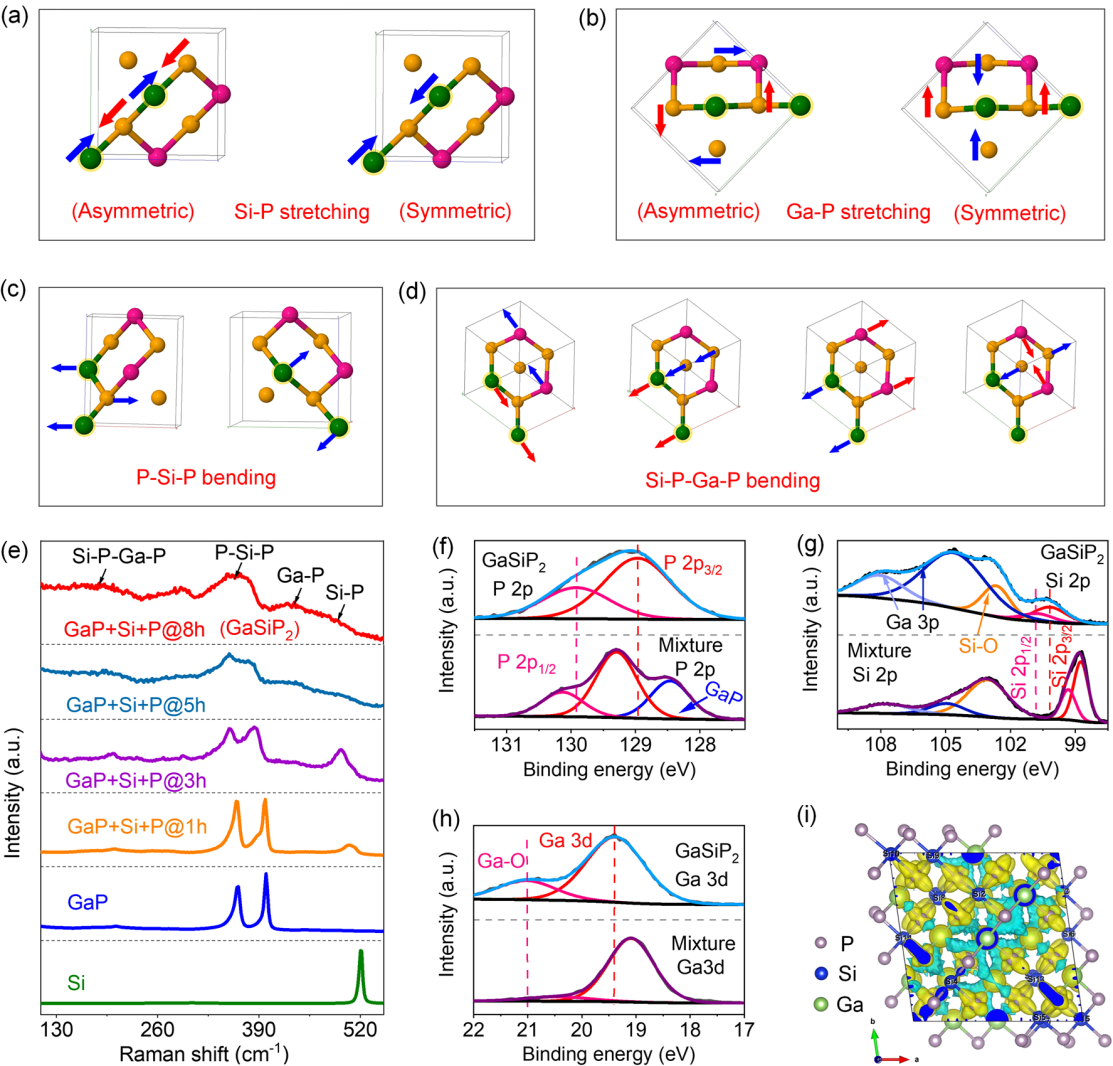
Raman vibration analysis of the cation-mixed GaSiP_2_: **a** Si–P, 495–470 cm^−1^; **b** Ga–P, 430–410 cm^−1^; **c** P–Si–P, 365–375 cm^−1^; **d** Si–P–Ga–P, 220–180 cm^−1^. (Nate that: the blue and red arrows represent the atomic movements). **e** Raman spectra of the cation-mixed GaSiP_2_ compound, ball-milled GaP + Si + P intermediates, GaP, and Si. High-resolution XPS spectra of the GaSiP_2_ and the mixture of GaP + Si + P: **f** P 2*p*; **g** Si 2*p*; **h** Ga 3*d*. **i** Charge density difference of the cation-mixed GaSiP_2_ compound

Electronic and chemical structures of the as-prepared cation-mixed GaSiP_2_ compound were characterized analyzing Raman and XPS spectra. As presented in Figs. 3a–e and S3, the vibration modes of the cation-mixed GaSiP_2_ compound were resolved. The theoretically calculated vibration frequencies in asymmetric and symmetric stretching modes of Si–P (Fig. 3a) and Ga–P (Fig. 3b) are located at 470–495 cm^−1^ and 410–430 cm^−1^, respectively, which is in a good agreement with experiment observation of 410 to 493 cm^−1^. The calculated vibration frequencies in bending modes of P–Si–P (Fig. 3c) and Si–P–Ga–P (Fig. 3d) skeletons are in the range from 365 to 375 cm^−1^ and from 220 to 180 cm^−1^, respectively, while those are experimentally observed in the range from 349 to 378 cm^−1^ and from 220 to 170 cm^−1^, respectively. As shown in Fig. 3e, Raman spectra of the as-synthesized GaSiP_2_ compound are substantially different from those of the ball milled intermediates of GaP + Si + P@*x*h, Si, and GaP. Thus, the experimental observations are supported by the calculation results, confirming the formation of the cation mixed GaSiP_2_ compound. As shown in Figs. 3f and S4, the XPS P 2*p* peak of the cation-mixed GaSiP_2_ compound shift to a lower binding energy than that of the P of the GaP + Si + P mixture, indicating that the phosphorus of the cation-mixed GaSiP_2_ compound is negatively charged. Moreover, the binding energy of P for the cation mixed GaSiP_2_ compound is higher than that of the GaP compound indicating its higher negative valence state. The Si 2*p* peak of the cation-mixed GaSiP_2_ compound is different from those of the GaP + Si + P mixture, which suggests different oxidation states (Fig. 3g). The relatively high Si valence state of Si–O originates from the surface oxidation of the as-prepared GaSiP_2_ compound, which is attributed to the inevitable exposure to air in the experiment process. The peak with higher binding energy comes from the Ga 3*p* instead of Si. The slightly positively charged state of Si 2*p* from the GaSiP_2_ compound proves a new compound formation. As shown in Fig. 3h, the Ga 3*d* signal of the cation mixed GaSiP_2_ compound owns slightly higher binding energy than that of the GaP compound due to the introduced Si and P which results in the redistribution of electrons. The capability to lose electrons of the metallic Ga is much higher than that of Si, thus leading to high binding energy (larger polarization) of Ga and relatively low binding energy (smaller polarization) of Si [46]. Moreover, the Ga–O signal of the cation-mixed GaSiP_2_ compound is attributed to the surface oxygen adsorption of the GaSiP_2_. The charge density difference of the cation mixed GaSiP_2_ compound is shown in Fig. 3i, and its constituent elements of Ga and Si are positively charged, while P is negatively charged, which is well-consistent with the XPS results. The concentrations of Ga, Si, and P elements within the cation-mixed GaSiP_2_ compound measured by ICP-OES are well consistent with the theoretical ones (Fig. S5). In particular, the cation-mixed lattice of the GaSiP_2_ compound is preserved after sintering in the air at 400 ºC for 24 h (Fig. S6). The above spectral characterizations confirm the successful synthesis of the ternary cation mixed GaSiP_2_ compound with the well-defined electronic and chemical structure.

### Li-storage Performances and Kinetics of Cation-mixed GaSiP_2_ Compounds

**Fig. 4:**
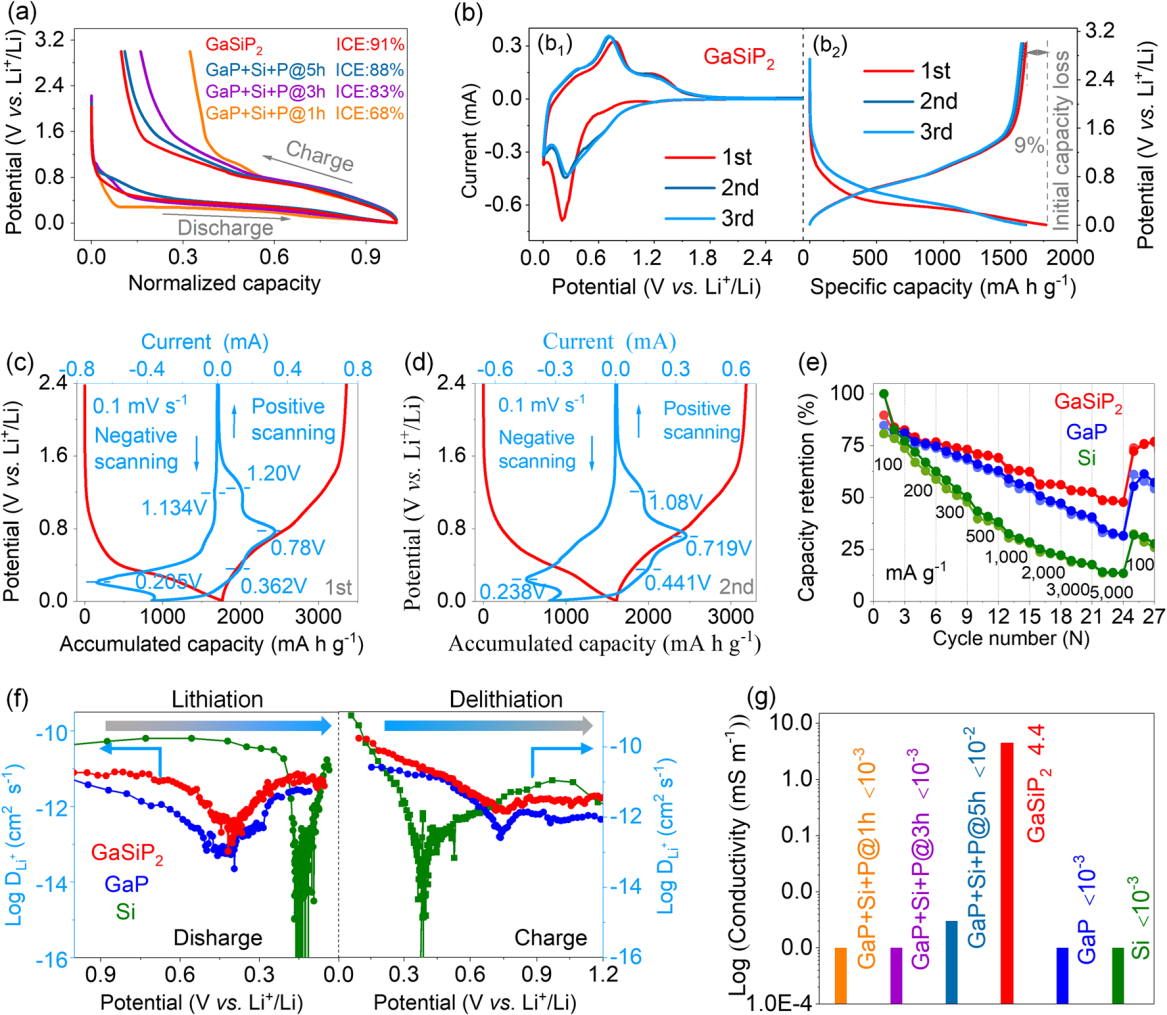
**a** First discharge and charge profiles of the GaSiP_2_ and ball milled intermediates of GaP + Si + P@*x*h samples at a current density of 100 mA g^−1^; **b** initial three CV curves at a scan rate of 0.1 mV s^−1^ and first three discharge and charge profiles. CV curves along with the discharge and charge profiles: **c** First; **d** Second. **e** Rate performance; **f** Li-ions diffusion coefficient of the GaSiP_2_, GaP, and Si. **g** Electronic conductivity of the cation-mixed GaSiP_2_ compound, the ball-milled intermediates of GaP + Si + P@*x*h, GaP, and Si

**Fig. 5:**
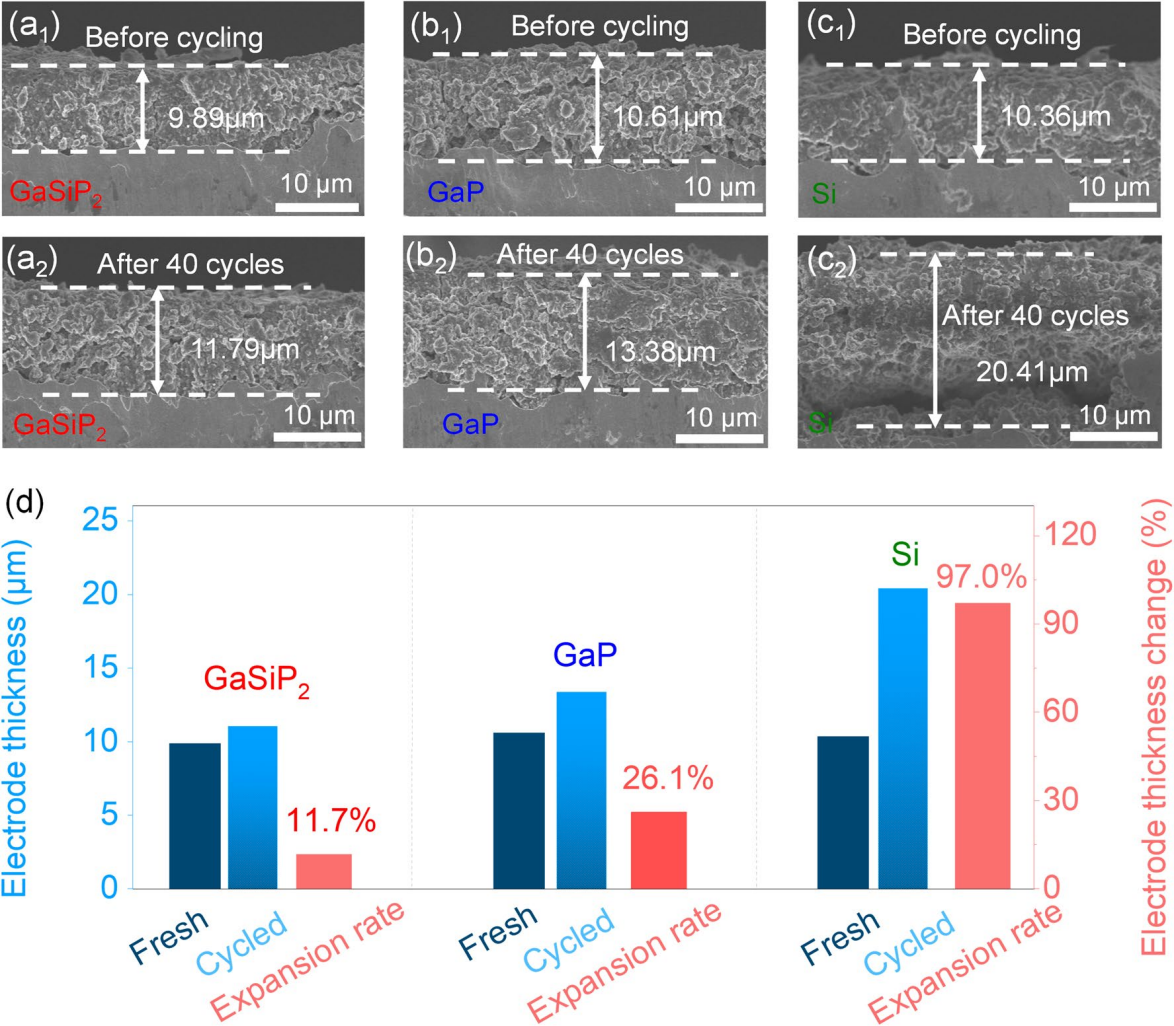
Cross-section images of the GaSiP_2_, GaP and Si: **a**_**1**_–**c**_**1**_ Pristine electrodes; **a**_**2**_–**c**_**2**_ Electrodes after 40 cycles; **d** Electrode thickness variation and expansion ratio

The lithium storage characteristics of the cation mixed GaSiP_2_ compound are investigated. As shown in Fig. 4a, the cation-mixed GaSiP_2_ compound shows smoother discharge and charge profiles with a higher ICE up to 91%, and smaller potential polarization, than those of the ball-milled intermediates of GaP + Si + P@*xh*. The initial three CV (Fig. 4b_1_) curves at 0.1 mV s^−1^ and GCD profiles (Fig. 4b_2_) show the onset reduction potential at about 1.134 V during the initial negative scanning process, representing the formation of the solid electrolyte interface (SEI) [47, 48]. The first reduction stage from 1.0 to 0.3 V corresponds to the initial Li-ion intercalation process by forming Li_*x*_GaSiP_2_ (*x* < 2, Fig. 4c), and the subsequent reduction peak at 0.205 V is assigned to the binary-alloys formation of P–Li, Si–Li, and Ga–Li arising from the decomposition of the above Li-intercalation compounds. Reversely, there are three oxidation peaks at 0.362, 0.780, and 1.200 V, indicating Li-ion extraction from the Ga–Li alloys along with Si–Li alloys, P–Li alloys, and Li_*x*_GaSiP_2_ (*x* < 2), respectively. As plotted in Fig. 4d, the second CV cycle is almost the same as the first one, demonstrating relatively smaller polarizations associated with the enhanced reaction kinetics by the lattice relaxation at the first cycle [49]. These CV features are well consistent with the initial three GCD profiles, which demonstrates the excellent electrochemical performances of the cation mixed GaSiP_2_ such as higher ICE and smoother GCD profiles during the first three cycles than those of the parent GaP and Si phases (Fig. S7a). No capacity of the cation mixed GaSiP_2_ compound was decayed after 80 cycles, which is superior to the ball milled intermediates of the GaP + Si + P@*x*h and its parent GaP and Si phases (Fig. S7b). Additionally, the GaSiP_2_ compound anode exhibits higher capacity retentions at different current densities (Fig. 4e). Especially, at a high rate of 5000 mA g^−1^, the GaSiP_2_ presents a capacity retention of 48% much higher than those of the parent GaP (33%) and Si (14%) phases. These superior performances are attributed to the faster Li-ionic and electronic conductivities and stronger resistance against volume variation of the cation-mixed GaSiP_2_ compound than its parent phases of GaP and Si, as calculated above (Fig. 1). Additional experimental measurements were performed to validate it. Firstly, GITT measurements were carried out to determine the diffusion behaviors and thermodynamics of Li-ions [28, 50–52]. Within the range of the operating potentials, the Li-ionic diffusion coefficient of the cation-mixed GaSiP_2_ compound is ~ 10^–12^ cm^2^ s^−1^, which is larger than those of the parent GaP and Si phases (Figs. 4f and S8) and the ball-milled intermediates of GaP + Si + P@*x*h (Figs. S9 and S10) during the first discharge and charge process. As demonstrated in Fig. 4g, the electronic conductivity of the cation-mixed GaSiP_2_ compound is 4.4 mS m^−1^, three magnitudes higher than those of its parent GaP and Si phases. Meanwhile, the single-phase GaSiP_2_ compound owns significantly higher (three magnitudes higher) electronic conductivity than its ball-milled intermediates of the GaP + Si + P@*x*h powders. Furthermore, we measured the electrochemical impedance spectra of the cation-disordered GaSiP_2_ compound, GaP and Si (Fig. S11). According to the equivalent circuit diagram (Fig. S11b), the charge transfer resistance (*R*_ct_) of GaSiP_2_ electrode is 82 Ω, which is much smaller than those of GaP (124 Ω) and Si (643 Ω).The *ex situ* SEM characterizations of the anode after cycling were performed as shown in Fig. 5. The cation mixed GaSiP_2_ compound shows the best tolerance against the volume variation among the GaSiP_2_, GaP, and Si anodes. In particular, the Ga-based compounds of GaSiP_2_ and GaP present no obvious cracks after 40 cycles in a different manner to Si anodes (Fig. 5a_2_–c_2_). Furthermore, the variation in the thickness of the GaSiP_2_ compound electrode is smaller than those of GaP and Si (Fig. 5d), which is most likely due to the self-healing ability of the liquid metal Ga element [22–24, 53].

### Li-storage Mechanisms of Cation-Mixed GaSiP_2_ Compounds

**Fig. 6:**
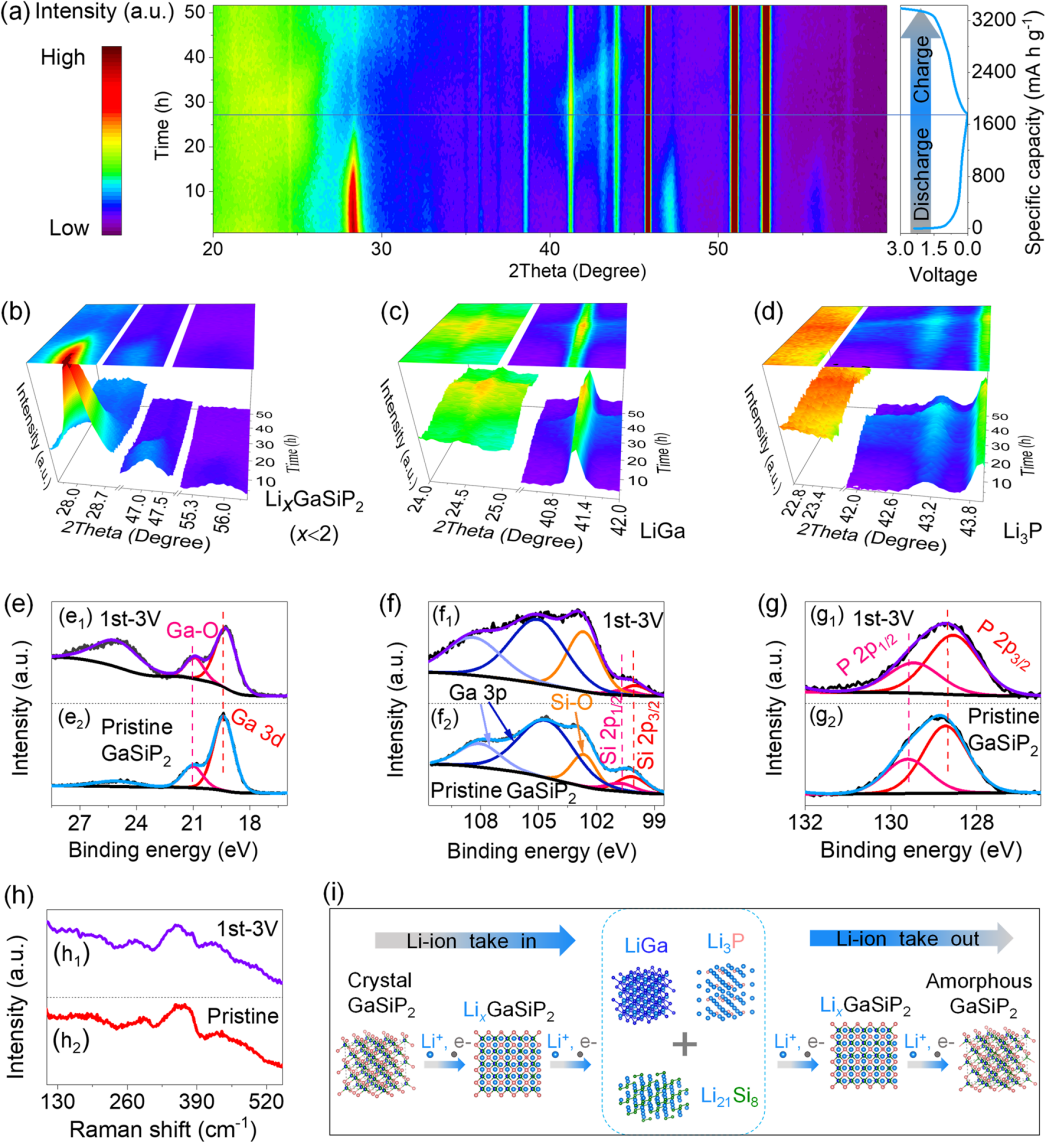
**a** *In situ* XRD contour image along with the discharge and charge profiles of the cation-mixed GaSiP_2_; **b**–**d** Evolution of the GaSiP_2_ compound, LiGa and Li_3_P; **e**–**g** *Ex situ* XPS; **h** *Ex situ* Raman. **i** Schematic illustration of the Li-storage mechanisms

The *in situ* XRD and *ex situ* XRD, Raman, and XPS measurements were performed to shed light on the Li-storage mechanisms of the as-prepared cation-mixed GaSiP_2_ compound. *In situ* XRD contour image of the cation-mixed GaSiP_2_ is given with the GCD profiles as shown in Fig. 6a. Upon the intercalation of Li-ions, the characteristic peaks of GaSiP_2_ shift to a lower 2theta showing a weaker intensity (Fig. 6b). Specifically, the shifts of characteristic peaks at the discharging voltage of 0.37 V are described by *ex situ* XRD (Figs. S12b_2_ and S13). This observation is attributed to the intercalation process of Li-ions, leading to the lattice expansion of the cation-mixed GaSiP_2_ compound. The shifting difference at different 2theta degrees is attributed to the anisotropy of the crystalline lattice. Along with more Li-ions intercalated, the characteristic peaks of the cation-mixed GaSiP_2_ compound completely disappeared, while the peaks of binary alloys of Li_3_P and LiGa gradually appeared (Figs. 6c–d and S12). In addition, the peak of Li_21_Si_8_ can be detected by *ex situ* XRD (Fig. S12b_5_). Compared with Li_22_Si_5_ (400%), Li_21_Si_8_ (170%) is more favorable for reducing the volume expansion of the cation mixed GaSiP_2_ compound despite sacrificing the specific capacity [4, 54, 55]. Both the Li-ion conductor of Li_3_P and electron conductor of LiGa are formed, leading to faster reaction kinetics of the electrode [56, 57]. Reversely, Li-ions were gradually retracted from the electrode upon charging, while the diffraction peaks for the binary alloy phases of Li_3_P, LiGa, and Li_21_Si_8_ gradually disappeared. When fully charged to 3.0 V, the electrode became amorphous, showing no obvious XRD peaks. To validate it, *ex situ* XPS characterizations of the electrode after charging were performed (Fig. S14). As illustrated in Fig. 6e–g, the slight shifts of Ga, Si, and P elements after charging to 3.0 V toward the lower binding energy compared to the pristine state can be attributed to the amorphization. Furthermore, as compared in Fig. 6h, the Raman spectra of the cation-mixed GaSiP_2_ compound appear again after cycling, which means that the Li-storage process is highly reversible. It notes that the amorphization of the electrode after the initial cycling is conductive to reduce the electrode fracturing upon cycling, due to an isotropic stress exerted on the amorphous structure [58, 59]. The Li-storage mechanisms during the lithiation and delithiation process are summarized as shown in a schematic illustration (Fig. 6i). According to the above-mentioned characterizations, the Li-ion storage mechanisms of the ternary cation-mixed GaSiP_2_ compound electrodes can be summarized as follows:

During the lithiation stage:

1 $${\text{GaSiP}}_{{2}} + x{\text{Li}}^{ + } + x{\text{e}}^{ - } \to {\text{Li}}_{x} {\text{GaSiP}}_{{2}} \;(x < {2})$$

2 $${\text{Li}}_{x} {\text{GaSiP}}_{{2}} + \left( {{9}.{625} - x} \right){\text{ Li}}^{ + } + \left( {{9}.{625} - x} \right){\text{ e}}^{ - } \to {\text{LiGa }} + {\text{Li}}_{{{2}.{625}}} {\text{Si}} + {\text{2Li}}_{{3}} {\text{P}}$$

During the delithiation stage:

3 $${\text{LiGa}} + {\text{Li}}_{{{2}.{625}}} {\text{Si}} + {\text{2Li}}_{{3}} {\text{P }} - \left( {{9}.{625} - x} \right){\text{ Li}}^{ + } - \left( {{9}.{625} - x} \right){\text{ e}}^{ - } \to {\text{Li}}_{x} {\text{GaSiP}}_{{2}}$$

4 $${\text{Li}}_{x} {\text{GaSiP}}_{{2}} - x{\text{Li}}^{ + } - x{\text{e}}^{ - } \to {\text{GaSiP}}_{{2}} \left( {{\text{amorphous}}} \right)$$

Overall reaction:

5 $${\text{GaSiP}}_{{2}} + {9}.{\text{625Li}}^{ + } + {9}.{\text{625e}}^{ - } \leftrightarrow {\text{LiGa}} + {\text{Li}}_{{{2}.{625}}} {\text{Si }} + {\text{ 2Li}}_{{3}} {\text{P}}$$

### Improved Li-storage Performances of the GaSiP_2_@C Composites

**Fig. 7:**
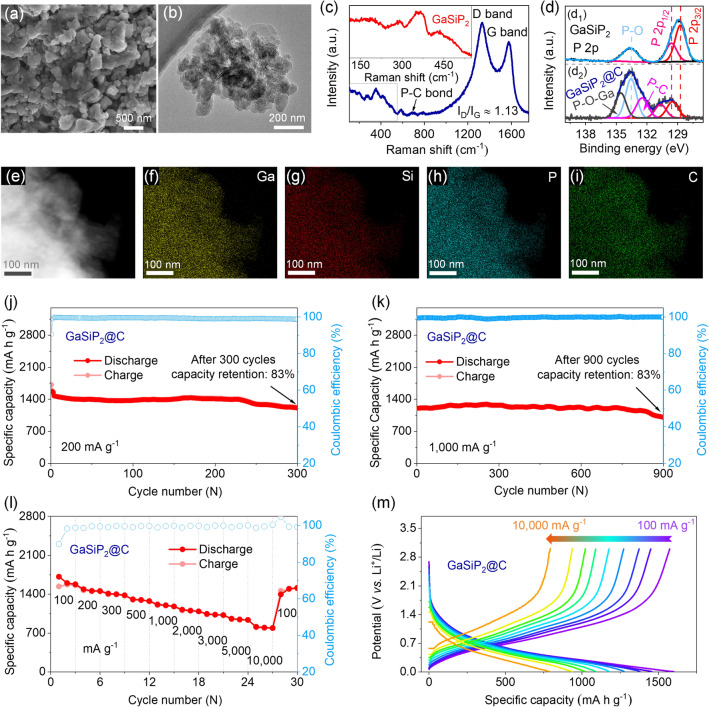
**a** FESEM image and **b** low-magnification TEM image of the GaSiP_2_@C composite. **c** Raman spectrum of the pristine cation-mixed GaSiP_2_ compound (the inset) and the GaSiP_2_@C composite; **d** High-resolution XPS spectra of P 2*p*; **e**–**i** Elemental mapping images; Capacity retentions **j** over 300 cycles at 200 mA g^−1^ and **k** over 900 cycles at 1,000 mA g^−1^, respectively; **l** Rate performance; **m** Discharge and charge curves at different current rates

For the enhancement of the cycling stability and rate performance of the anode, the ternary cation mixed GaSiP_2_ compound was blended with graphite to construct GaSiP_2_@C composite at the mass ratio of 6: 3 (GaSiP_2_: graphite) via a secondary ball milling process. As shown in the TGA curves at temperatures from 25 to 1200 °C (Fig. S15), the carbon content of GaSiP_2_@C is about 32.0%, which is well-consistent with the mass ratio of graphite (33.33%) in the composite. And the distribution of GaSiP_2_ is quite uniform as shown in Fig. 7a–b. In Fig. 7c, the intensity of D band (disordered *sp*^3^ C–C) at 1332 cm^−1^ is higher than that of the G band (ordered *sp*^2^ C = C) at 1577 cm^−1^ [60, 61], indicating that some of the graphitic layers were destroyed into amorphous carbon uniformly dispersing the nanosized GaSiP_2_ compound. According to the empirical Tuinstra-Koenig equation, La = (2.4 × 10^–10^) *λ*^4^ (*I*_D_/*I*_G_)^−1^ (*λ* = 532 nm) [62, 63], the average grain size is calculated as 17.1 nm. The small grains are conducive to provoke both easy electrolyte penetration and short Li-ion diffusion length [64]. In particular, the new broad Raman characteristic peak at 600–700 cm^−1^ appeared for the GaSiP_2_@C composite due to the P–C bonds formed at the interface between GaSiP_2_ and graphite. Moreover, the existence of the P–C bond was further confirmed by the XPS analyses (Fig. 7d) [65, 66]. These P–C bonds contribute to improve Li-ionic and electronic conductivities and to depress the volume variation upon charging and discharging [30]. As verified by HAADF-STEM images of the GaSiP_2_@C composite in Fig. 7e–i, the elements of Ga, Si, P, and C are well distributed without any segregation, indicating the uniform dispersion of the GaSiP_2_ compound within the carbon matrix. The electronic conductivity of the GaSiP_2_@C composite is about 3 orders of magnitude higher than that of the pristine GaSiP_2_ compound (Fig. S16).

The GaSiP_2_@C composite electrode achieved the capacity retention of 83% after 300 cycles at a low current density of 200 mA g^−1^ (Figs. 7j and S17a). Moreover, the cycling stability of the as-prepared GaSiP_2_@C composite was confirmed demonstrating the capacity retention rate of 83% after 900 cycles at a high current rate of 1,000 mA g^−1^ in the working potential range of 3.000 to 0.005 V (Figs. 7k and S17b). The enhanced rate performance is also observed, as shown in Fig. 7l–m. The GaSiP_2_@C electrode delivered the high specific capacities of 1452 to 941 mAh g^−1^ varying the current densities at 200 to 5000 mA g^−1^, achieving 65% of capacity retention. Even at a high current density of 10,000 mA g^−1^, the electrode still maintained the high-rate capacity of 800 mAh g^−1^. As the current density returned to the initial value of 100 mA g^−1^, the initial reversible capacity was recovered. As presented by Table S5, the long-term cycling stability and rate capability of the GaSiP_2_@C composite are comparable with or superior to those of other Ga-based, Si-based, and P-based anode materials previously reported. In particular, the LiNi_0.8_Co_0.1_Mn_0.1_O_2_//GaSiP_2_@C full cells deliver the high specific capacity of 1,049 mAh g^−1^ after 100 cycles in the working potential of 0.5–4.1 V (Fig. S18). The superior Li-storage performance of the cation-mixed GaSiP_2_@C composite is attributed to the uniform distribution of the cation-mixed GaSiP_2_ compound within the carbon matrix, where P–C bonds are formed at the interface, making it promising for practical applications.

## Conclusions

In summary, we have demonstrated the cation-mixed disordered lattice and self-healing liquid metal Ga incorporation of single-phase ternary GaSiP_2_ compound. The crystalline structure, electronic structure, and mechanical and electrochemical properties of GaSiP_2_ compound were comprehensively analyzed through experimental characterizations and first-principles calculations. The introduction of liquid metallic Ga phase and reactive P and the cation-mixed disordered lattice allowed the GaSiP_2_ electrode to achieve stronger resistance to electrode pulverization, higher metallic conductivity, and faster Li-ionic diffusion than those of GaP and Si counterparts. In particular, the GaSiP_2_ anode delivered a reversible capacity of 1615 mAh g^−1^ with an ICE of 91%, and a suitable working potential of 0.45 V through the self-healing Li-storage mechanism based on the intercalation and subsequent conversion reaction as analyzed by the *in situ*/*ex situ* spectroscopic methods. Furthermore, the GaSiP_2_@C composite electrode achieved excellent long-term cycling stability with a capacity retention ratio of 83% after 900 cycles at a current density of 1000 mA g^−1^ and a high-rate capability with a capacity of 800 mAh g^−1^ at 10,000 mA g^−1^, thus promising to be new-generation anode materials candidates for LIBs.

### Supplementary Information

Below is the link to the electronic supplementary material.

Supplementary file1 (PDF 2963 KB)

Supplementary file2 (MP4 258 KB)
