# Supplementary material for: Single-Phase Ternary Compounds with a Disordered Lattice and Liquid Metal Phase for High-Performance Li-Ion Battery Anodes
Source: Nanomicro Lett. 2023 Mar 10;15:63. doi: 10.1007/s40820-023-01026-4 (PMC10006393; doi:10.1007/s40820-023-01026-4)
Supplement: Supplementary file 1 — Supplementary file1 (PDF 2963 KB) [file 40820_2023_1026_MOESM1_ESM.pdf]

Supporting Information for

Single-Phase Ternary Compounds with a Disordered Lattice and Liquid Metal Phase for High Performance Li-Ion Battery Anodes

Yanhong Li[1,2], Lei Zhang[3], Hung-Yu Yen[4], Yucun Zhou[3], Gun Jang[1], Songliu Yuan[2], Jeng-Han Wang[4], Peixun Xiong[1], Meilin Liu[3], Ho Seok Park[1,5,6,7,*], and Wenwu Li[1,*]

[1] School of Chemical Engineering, Sungkyunkwan University, 2066, Seobu-ro, Jangan-gu, Suwon-si, Gyeonggi-do, 440-746, Korea

[2] School of Physics, Huazhong University of Science and Technology, Wuhan, 430074, P. R. China

[3] School of Materials Science & Engineering, Georgia Institute of Technology, Atlanta, GA 30332, USA

[4] Department of Chemistry, National Taiwan Normal University, Taipei, 11677, Taiwan, P. R. China

[5] Samsung Advanced Institute for Health Sciences and Technology (SAIHST), Sungkyunkwan University, 2066 Seoburo, Jangan-gu, Suwon 440-746, Korea

[6] SKKU Advanced Institute of Nano Technology (SAINT), Sungkyunkwan University, 2066 Seoburo, Jangan-gu, Suwon 440-746, Korea

[7] SKKU Institute of Energy Science and Technology (SIEST), Sungkyunkwan University, 2066 Seoburo, Jangan-gu, Suwon 440-746, Korea

*Corresponding authors. E-mail: phs0727@skku.edu (Ho Seok Park); wenwuli@skku.edu (Wenwu Li)

Supplementary Figures and Tables

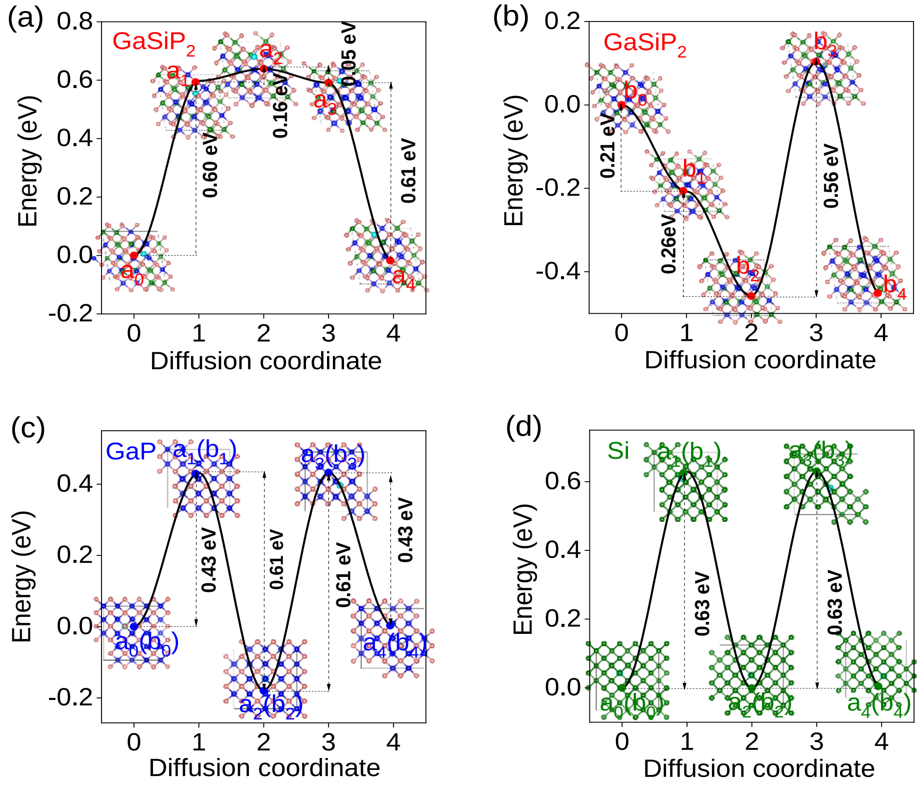

Fig. S1 Li-ionic diffusion barrier energies: **a** and **b** the cation-mixed $GaSiP_2$; **c** GaP; and **d** Si. Note: In terms of diffusion coordinates, the "1" and "3" are transition states and the "0", "2", and "4" are stable states

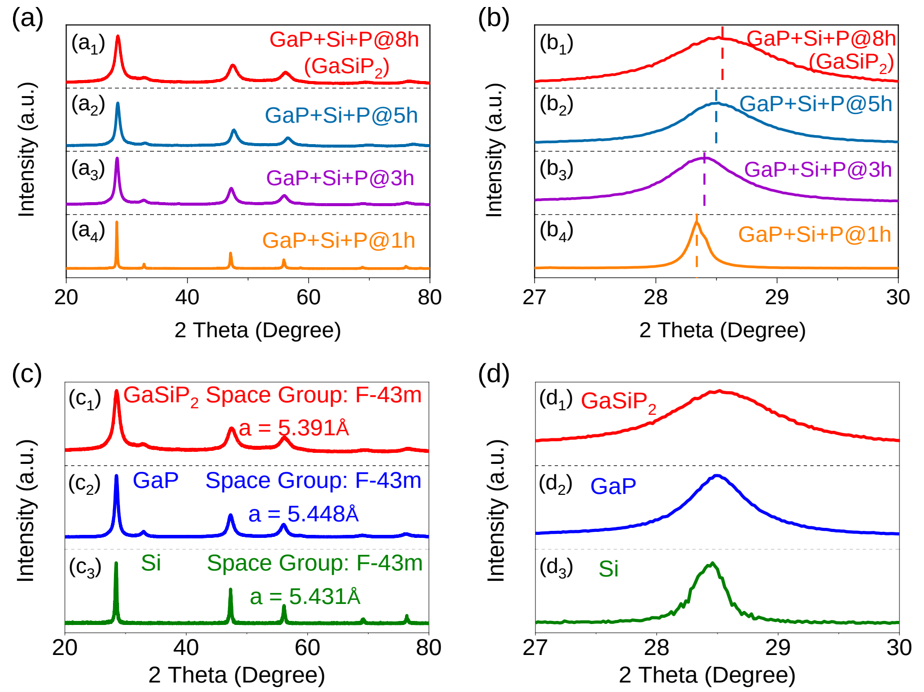

Fig. S2 a Change of the X-ray diffraction (XRD) patterns and **b** the corresponding XRD main peak of the ball-milled intermediates GaP+Si+P@xh mixture, in which "x" refers to the ball milling at "x" h; **c** the XRD patterns and **d** comparison of the main peak of the cation-mixed $GaSiP_2$, GaP and Si, respectively

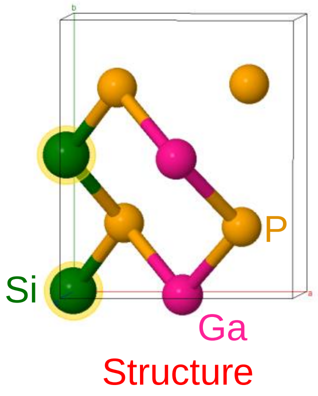

Fig. S3 $GaSiP_2$ supercell for Raman analysis

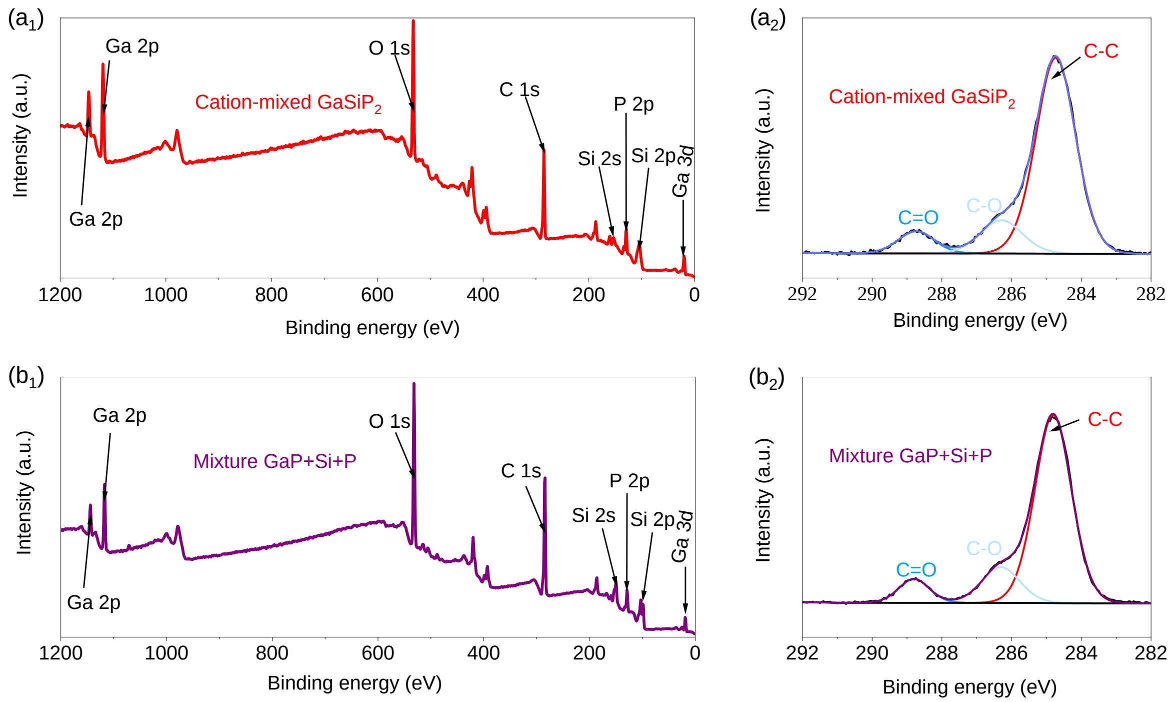

Fig. S4 High-resolution XPS spectra of full spectrum and C 1s: **a** Cation-mixed $GaSiP_2$ compound; **b** GaP+Si+P mixture

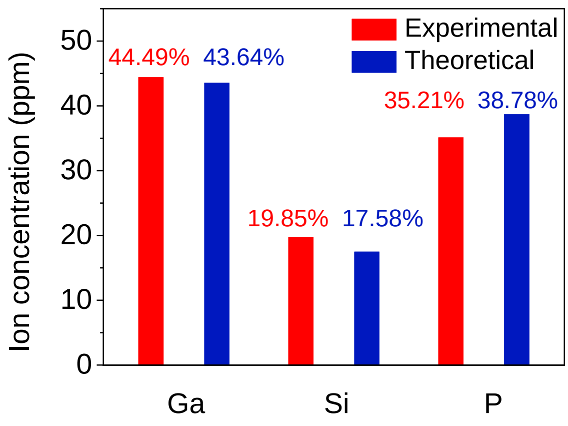

Fig. S5 Element concentrations of the cation-mixed $GaSiP_2$ compound measured by Inductively Coupled Plasma Optical Emission Spectrometer (ICP-OES)

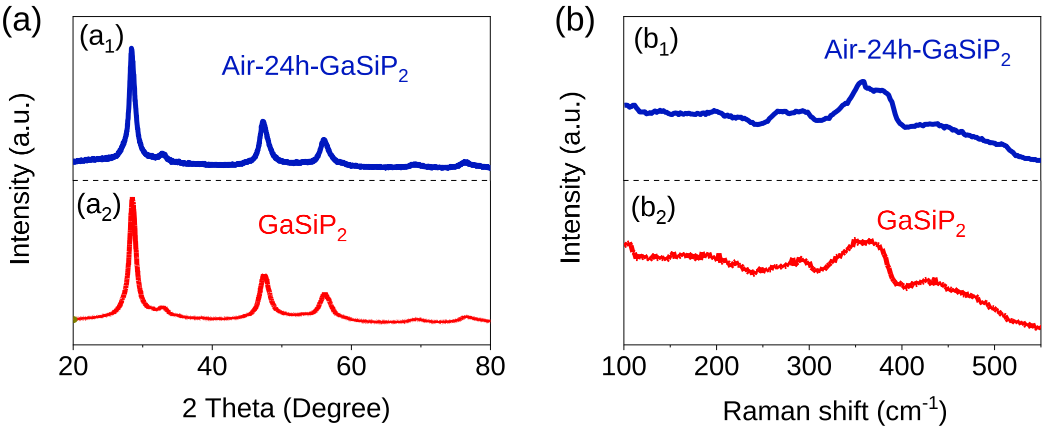

Fig. S6 a XRD; **b** Raman spectrum of the $GaSiP_2$ compound and after sintered at 400 °C for 24 h in the air. The unchanged signals indicate the stability of the cation-mixed $GaSiP_2$ compound

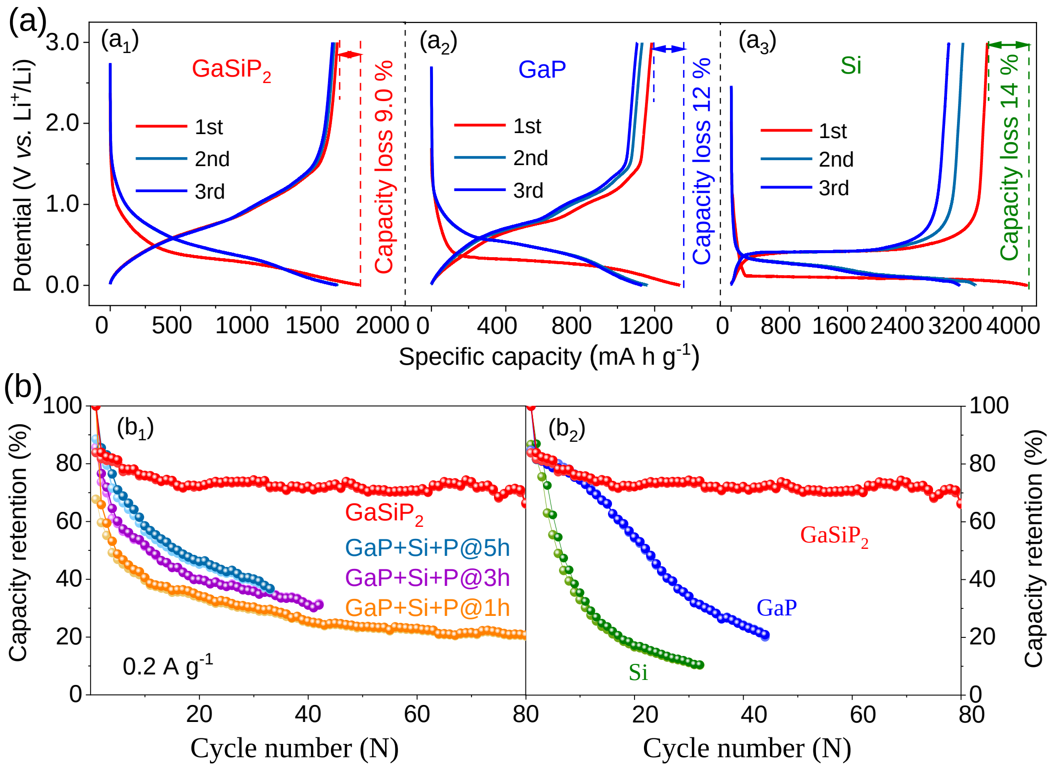

Fig. S7 Electrochemical characterizations of the cation-mixed $GaSiP_2$ compound, GaP, Si and the GaP+Si+P mixture: **a** First three discharge and charge profiles; **b** Cycle performance

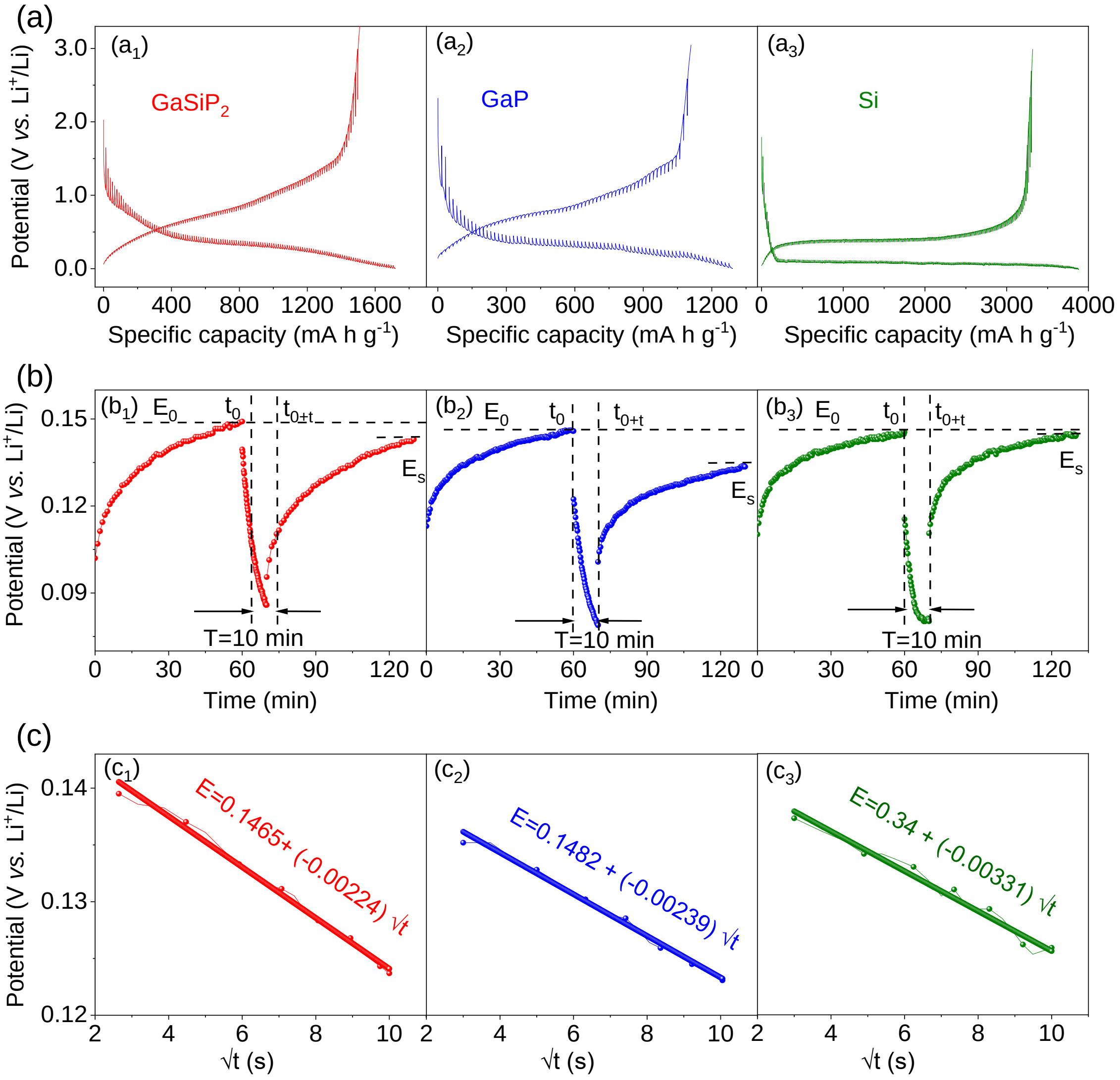

Fig. S8 Supplementary information of the galvanostatic intermittent titration technique (GITT) of the cation-mixed $GaSiP_2$, GaP and Si: **a** Voltage *vs.* capacity curves achieved by imposing a pulse time of 10 min and followed by a relaxation time of 1 h at a current density of 0.1 A g^{-1}; **b** Detailed voltage response over time during a single step of GITT test; and **c** Linear fit of the voltage change with the √t in the first 100 seconds

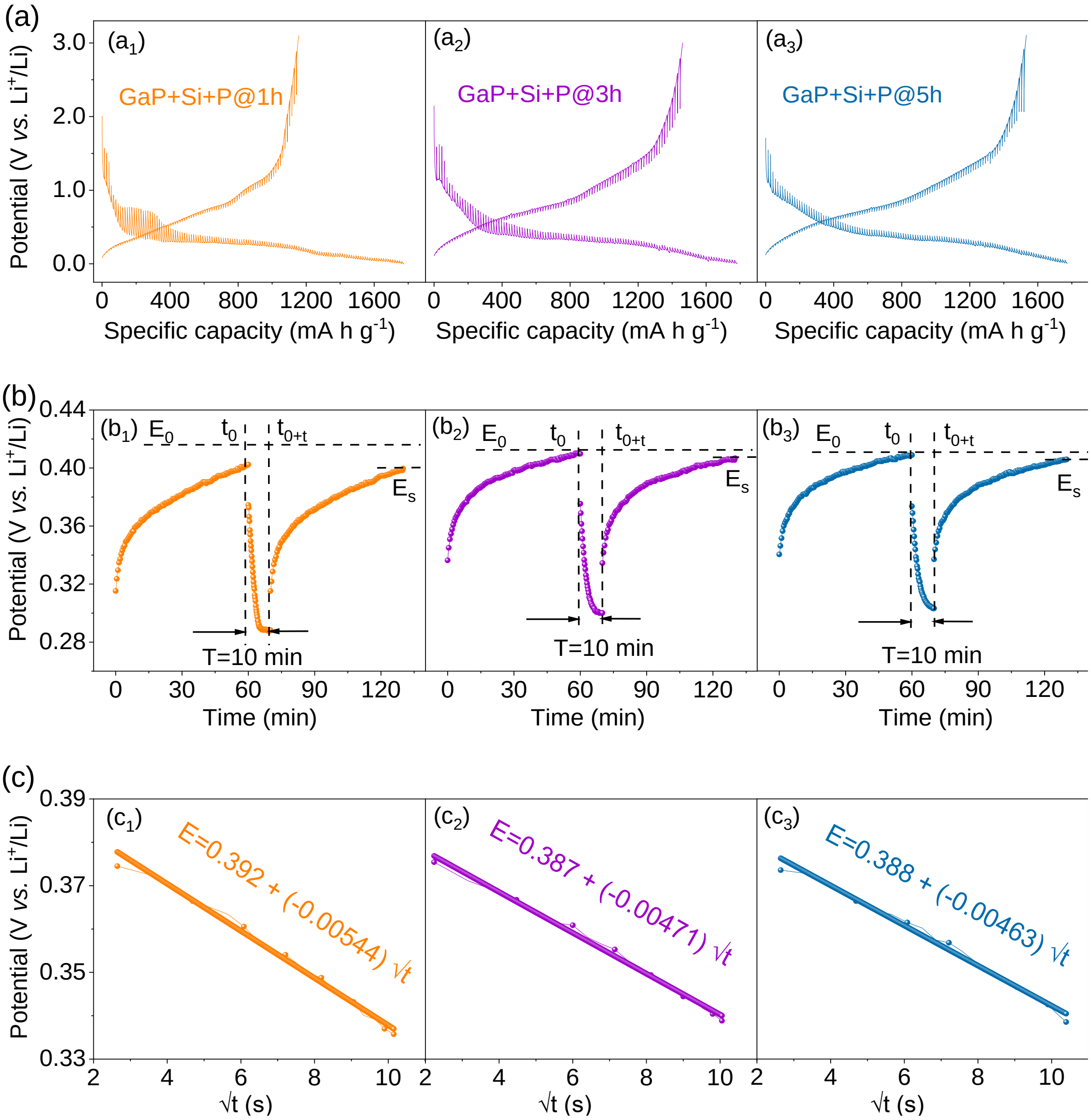

Fig. S9 Supplementary information of GITT in the ball milled intermediates: **a** Voltage *vs*. capacity curves achieved by imposing a pulse time of 10 min and followed by a relaxation time of 1 h at a current density of 100 mA g^{-1}; **b** Detailed voltage response over time during a single step of GITT test; and **c** Linear fits of the potential change with the √t in the first 100 seconds

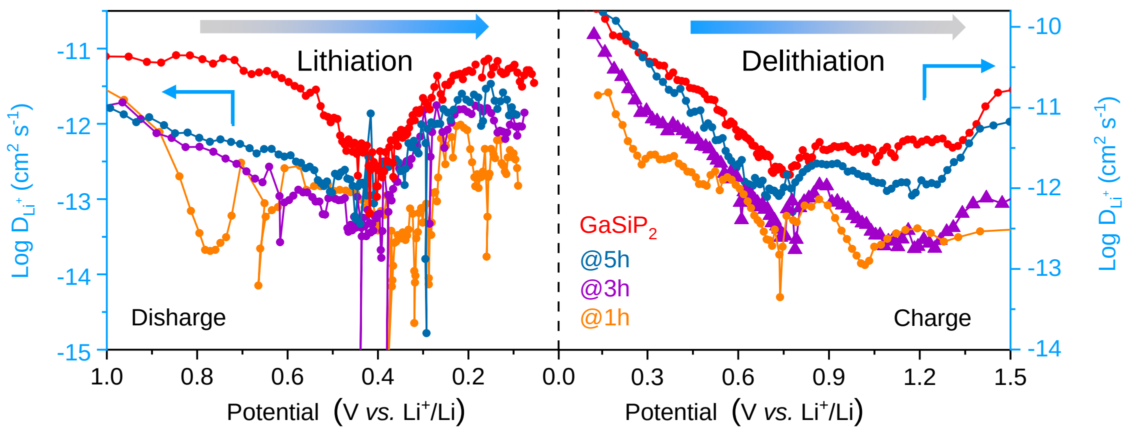

Fig. S10 Li-ionic diffusion coefficients of the cation-mixed $GaSiP_2$ and the corresponding ball milled intermediates during the first discharge and charge process

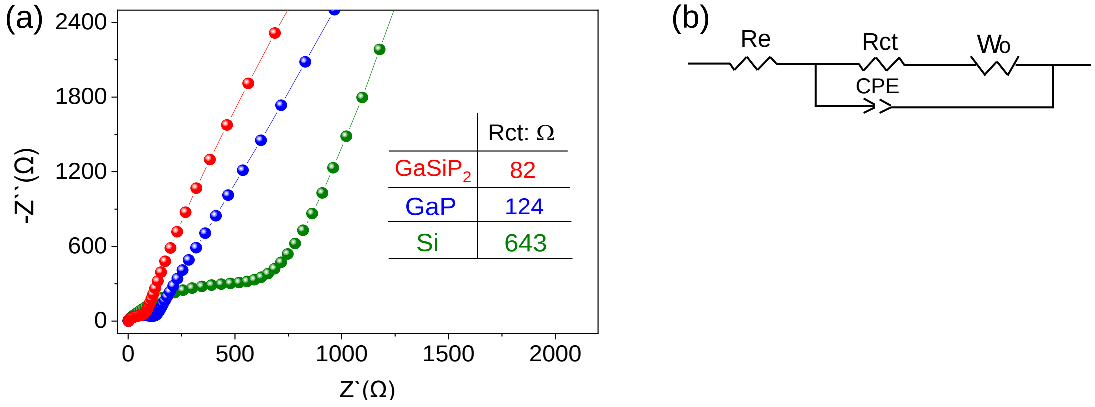

Fig. S11 **a** Electrochemical impedance spectroscopy of the cation-disordered $GaGeP_2$ compound, GaP, and Si. **b** Equivalent circuit diagram

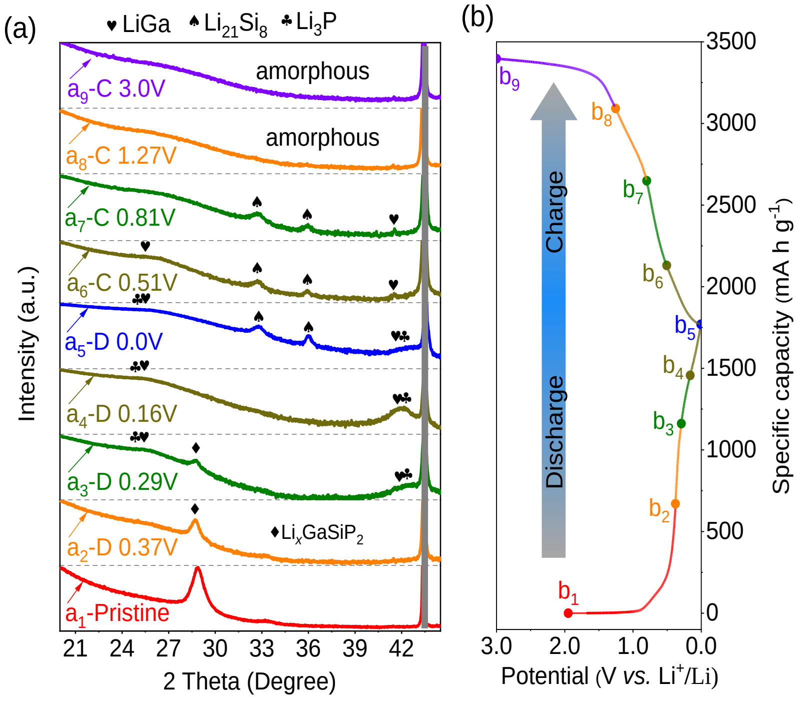

Fig. S12 **a** *Ex-situ* XRD along with **b** the selected working potentials

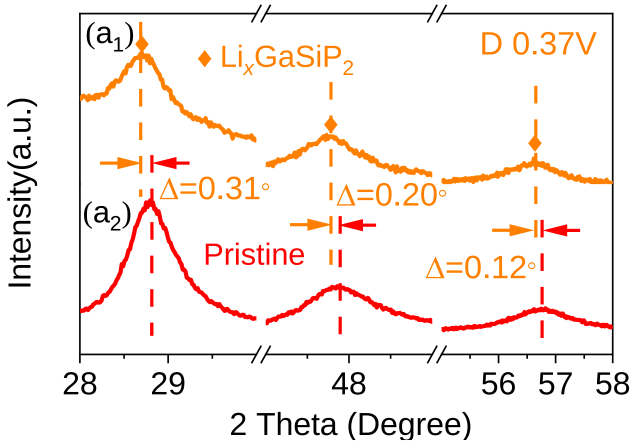

Fig. S13 *Ex-situ* XRD patterns of the pristine $GaSiP_2$ and Li_xGaSiP_2

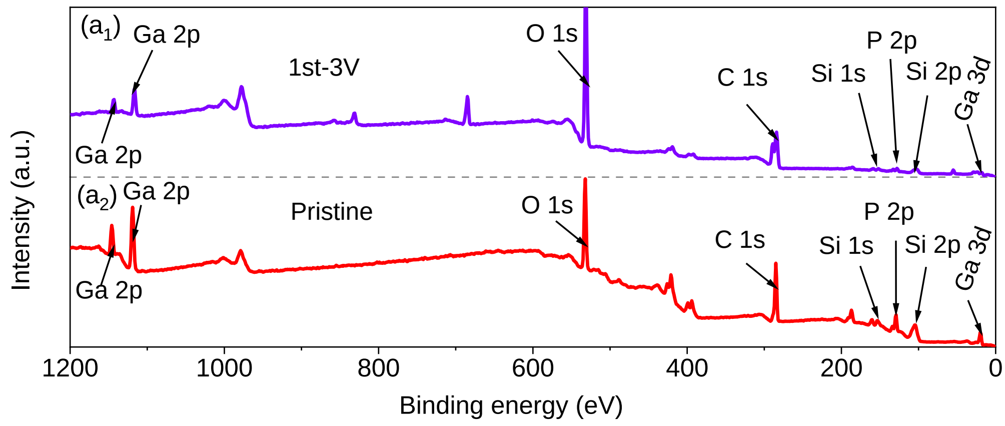

Fig. S14 High-resolution XPS spectra of full spectrum

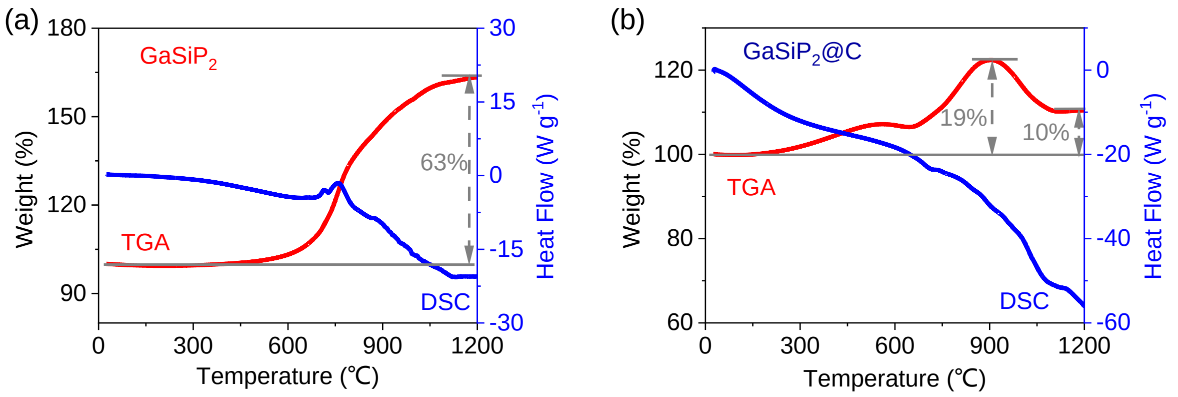

Fig. S15 Thermogravimetric analysis (TGA) curves: **a** Pristine $GaSiP_2$ compound; **b** $GaSiP_2$@C composite

Worth noting that the carbon content is calculated based on the following equations:

Assume that the carbon content in the composite is x and $GaSiP_2$ is y.

$$x + y = 1 \qquad (S1)$$

$$0.97y = 0.66 \qquad (S2)$$

Based on the above equations, the carbon content (x) is about 32.0% and consistent with the composition ratio of the two materials.

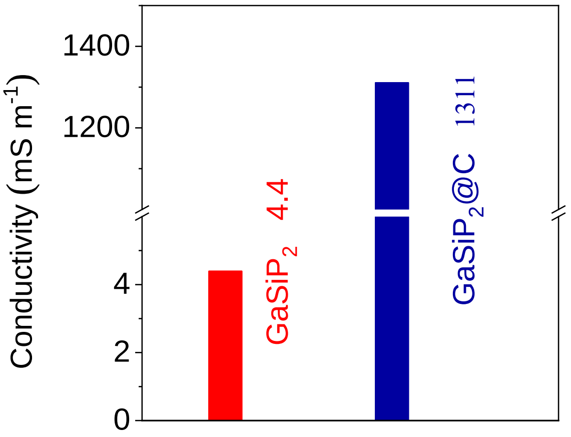

Fig. S16 Electronic conductivity of the pristine cation-mixed $GaSiP_2$ compound and the $GaSiP_2$@C composite

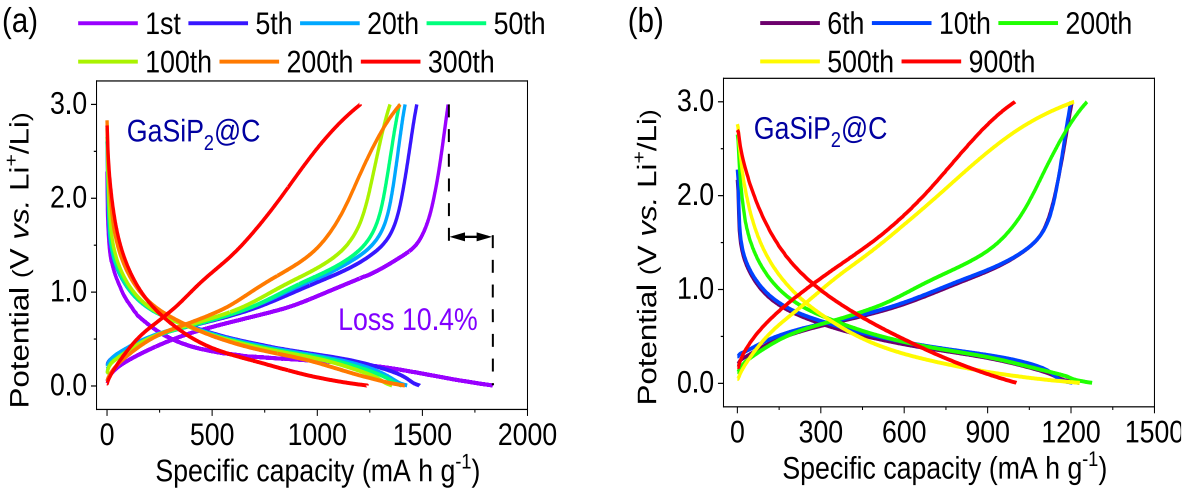

Fig. S17 a Discharge and charge profiles of the $GaSiP_2$@C, which is related to the **Fig. 7j**. (Note that: 1st at 100 mA g^{-1}, from 5th to 300th at 200 mA g^{-1}) **b** Discharge and charge profiles of the $GaSiP_2$@C at 1,000 mA g^{-1}, which is related to the **Fig. 7k**.

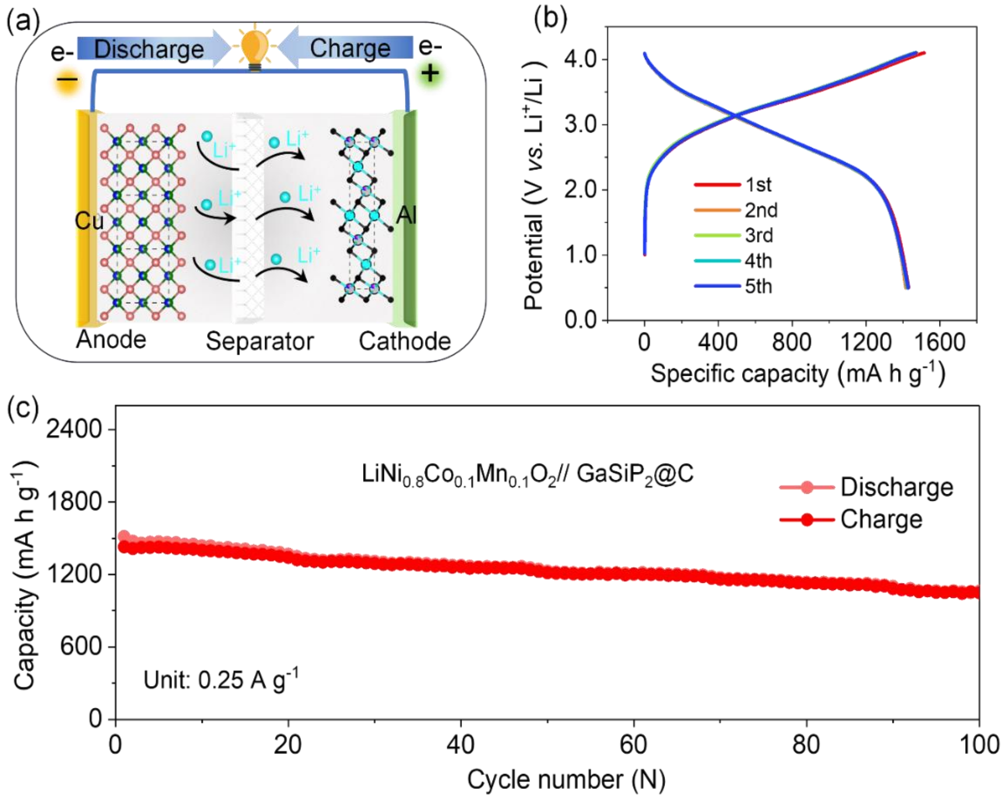

Fig. S18 Full cells assembled by the $LiNi_{0.8}Co_{0.1}Mn_{0.1}O_2$ cathode and the $GaSiP_2$@C composite anode: **a** Schematic image of the full cell; **b** Typical charge and discharge profiles; **c** Cycle performance

Table S1 Li-ionic diffusion barrier energies of the cation-disordered compound of $GaSiP_2$, GaP, and Si

Mater. / Path	$\mathbf{GaSiP_2}$	**GaP**	**Si**
$\mathbf{a_0 \rightarrow a_2}$	0.76	0.43	0.63
$\mathbf{a_2 \rightarrow a_4}$	0	0.61	0.63
$\mathbf{b_0 \rightarrow b_2}$	0	0.43	0.63
$\mathbf{b_2 \rightarrow b_4}$	0.56	0.61	0.63
**Average**	**0.33**	**0.52**	**0.63**

Table S2 Elastic constants of the cation-mixed $GaSiP_2$, GaP and Si

Mater. / Direction	**$GaSiP_2$**	**GaP**	**Si**
XX-XX	1386.7933	1343.8246	1570.336
YY-YY	1441.7788	1369.3133	1570.353
ZZ-ZZ	746.8587	1370.8395	1570.356
XY-XY	504.3055	678.6396	733.9069
YZ-YZ	527.4755	680.4182	736.0242
ZX-ZX	554.5123	680.8765	735.7903
**Average**	**860.2874**	**1020.652**	**1152.794**

Table S3 Fractional atomic coordinates and isotropic displacement parameter of the as-synthesized $GaSiP_2$ compound

	**x**	**y**	**z**	B_{iso}	**Occ.**
P	0.25	0.25	0.25	0.02401	1.0
Si	0	0	0	0.01266	1/2
Ga	0	0	0	0.12614	1/2

Table S4 Main parameters of processing and refinement of the $GaSiP_2$ sample

**Compound**	**$GaSiP_2$**
Crystal System	Cubic
Space Group	F-43m
V, Å^3	156.678
a, Å	5.391
2θ-interval	10-120
Z	8
Rwp%	4.36
Rp%	3.45
χ^2	2.62

Table S5 Comparisons of initial Coulombic efficiency (ICE), cyclic stability and rate performance of the cation-mixed $GaSiP_2$@C composite with the recently reported Ga-based, Si-based, and P-based anodes

Materials	ICE	Cycle performance	Rate performance	References
**$GaSiP_2$@C**	**90%**	**1.0 A g^{-1}, 900 cycles, 998 mA h g^{-1}**	**10 A g^{-1}, 800 mA h g^{-1}**	**This Work**
**Ga_2O_3-nanorods**	60%	1 .0 A g^{-1}, 400 cycles, 300 mA h g^{-1}	1.0 A g^{-1}, 200 mA h g^{-1}	[S1]
**Ga_2S_3-PAM**	68%	0.1 A g^{-1}, 200 cycles, 918 mA h g^{-1}	2.0 A g^{-1}, 474 mA h g^{-1}	[S2]
**Ga/Ga_2O_3@C**	59%	1 .0 A g^{-1}, 200 cycles, 542 mA h g^{-1}	5.0 A g^{-1}, 192 mA h g^{-1}	[S3]
**$CuGaS_2$ nanoplates**	69%	5.0 A g^{-1}, 600 cycles, 521 mA h g^{-1}	2.0 A g^{-1}, 621 mA h g^{-1}	[S4]
**GaN nanowires**	73%	10.0 A g^{-1}, 1000 cycles, 370 mA h g^{-1}	10.0 A g^{-1}, 256 mA h g^{-1}	[S5]
**LMNPs@CS fibers**	67%	1.0 A g^{-1}, 1500 cycles, 552 mA h g^{-1}	2.0 A g^{-1}, 499 mA h g^{-1}	[S6]
**$CuGa_2$**	81%	2.0 A g^{-1}, 60 cycles, 543 mA h g^{-1}	4.0 A g^{-1}, 463 mA h g^{-1}	[S7]
**BPQD/TNS**	42%	1.0 A g^{-1}, 2400 cycles, 520 mA h g^{-1}	2.0 A g^{-1}, 167 mA h g^{-1}	[S8]
**L-BP-Shexane**	40%	1.0 A g^{-1}, 200 cycles, 404 mA h g^{-1}	5.0 A g^{-1}, 273 mA h g^{-1}	[S9]
**SiOx–TiO_2@C**	63%	0.1 A g^{-1}, 600 cycles, 910 mA h g^{-1}	6.4 A g^{-1}, 372 mA h g^{-1}	[S10]
**Si-CG**	84%	8.4 A g^{-1}, 700 cycles, 1500 mA h g^{-1}	8.4 A g^{-1}, 737 mA h g^{-1}	[S11]
**BP@CNTs**	90%	0.1 A g^{-1}, 100 cycles, 750 mA h g^{-1}	2.5 A g^{-1}, 380 mA h g^{-1}	[S12]
**SiOx/G/C**	84%	0.3 A g^{-1}, 500 cycles, 524 mA h g^{-1}	3.0 A g^{-1}, 524 mA h g^{-1}	[S13]
**SiOC/Sb**	59%	0.372 A g^{-1}, 200 cycles, 530 mA h g^{-1}	2.232 A g^{-1}, 549 mA h g^{-1}	[S14]
**C-SiOx@Si/rGO**	71%	1.0 A g^{-1}, 100 cycles, 925 mA h g^{-1}	8.0 A g^{-1}, 906 mA h g^{-1}	[S15]
**Si@graphene cage**	55%	1.0 A g^{-1}, 200 cycles, 900 mA h g^{-1}	5.0 A g^{-1}, 890 mA h g^{-1}	[S16]

Supplementary Notes

The calculation method of the lithium diffusion coefficient:

The data were collected by using galvanostatic intermittent titration technique (GITT) [S17-S19]. Specifically, in the process of GITT measurement, a short pulse of 0.1 A g^{-1} was applied for 10 min, which is followed by a relaxation process of 1 h. The Li-ionic diffusion coefficients can be calculated according to the transient voltage responses during GITT tests by using the

following formula: D= $\frac{4}{\pi}\left(\frac{iV_m}{Z_AFS}\right)^2\left(\frac{\Delta Es}{\Delta Et}\right)^2$, in which, D stands for the Li-ionic diffusion coefficient ($cm^2\ s^{-1}$), i is the current (A) in the process of testing; V_m represents the molar volume of the electrode ($cm^3\ mol^{-1}$); Z_A is the charge number of Li-ion; F is the faraday constant (96485 C mol^{-1}); S is surface area of active materials; ΔEs stands for the steady-state voltage change through the current pulse, ΔEt is the voltage change with a current pulse after deducing IR drop.

Supplementary References

[S1] G. Meligrana, W. Lueangchaichaweng, F. Colò, M. Destro, S. Fiorilli et al., Gallium oxide nanorods as novel, safe and durable anode material for Li- and Na-ion batteries. Electrochim. Acta **235**, 143 (2017). https://doi.org/10.1016/j.electacta.2017.03.047

[S2] K. Wang, W. Ye, W. Yin, W. Chai, Y. Rui et al., A novel carbon-coated Ga_2S_3 anode material derived from post-synthesis modified MOF for high performance lithium ion and sodium ion batteries. Electrochim. Acta **322**, 134790 (2019). https://doi.org/10.1016/j.electacta.2019.134790

[S3] K. Wang, W. Ye, W. Yin, W. Chai, B. Tang et al., One-step synthesis of MOF-derived Ga/Ga_2O_3@C dodecahedra as an anode material for high-performance lithium-ion batteries. Dalton Trans. **48**, 12386 (2019). https://doi.org/10.1039/C9DT02651G

[S4] Y. Song, Y. Li, L. Zhu, Z. Pan, Y. Jiang et al., $CuGaS_2$ nanoplates: a robust and self-healing anode for Li/Na ion batteries in a wide temperature range of 268–318 K. J. Mater. Chem. A **6**, 1086-1093 (2018). https://doi.org/10.1039/C7TA09197D

[S5] C. Sun, X. Tang, Z. Yin, D. Liu, Y.-J. Wang et al., Self-supported GaN nanowires with cation-defects, lattice distortion, and abundant active sites for high-rate lithium-ion storage. Nano Energy **68**, 104376 (2020). https://doi.org/10.1016/j.nanoen.2019.104376

[S6] J. Zhu, Y. Wu, X. Huang, L. Huang, M. Cao et al., Self-healing liquid metal nanoparticles encapsulated in hollow carbon fibers as a free-standing anode for lithium-ion batteries. Nano Energy **62**, 883-889 (2019). https://doi.org/10.1016/j.nanoen.2019.06.023

[S7] Y. Shi, M. Song, Y. Zhang, C. Zhang, H. Gao et al., A self-healing $CuGa_2$ anode for high-performance Li ion batteries. J. Power Sources **437**, 226889 (2019). https://doi.org/10.1016/j.jpowsour.2019.226889

[S8] R. Meng, J. Huang, Y. Feng, L. Zu, C. Peng et al., Black phosphorus quantum dot/Ti_3C_2 MXene nanosheet composites for efficient electrochemical lithium/sodium-ion storage. Adv. Energy Mater. **8**, 1801514 (2018). https://doi.org/10.1002/aenm.201801514

[S9] W. Zheng, J. Lee, Z.W. Gao, Y. Li, S. Lin et al., Laser-assisted ultrafast exfoliation of back phosphorus in liquid with tunable thickness for Li-ion batteries. Adv. Energy Mater. **10**, 1903490 (2020). https://doi.org/10.1002/aenm.201903490

[S10] Z. Li, H. Zhao, P. Lv, Z. Zhang, Y. Zhang et al., Watermelon-like structured SiO_x–TiO_2@C nanocomposite as a high-performance lithium-ion battery anode. Adv. Funct. Mater. **28**, 1605711 (2018). https://doi.org/10.1002/adfm.201605711

[S11] L. Wang, T. Liu, X. Peng, W. Zeng, Z. Jin et al., Highly stretchable conductive glue for high-performance silicon anodes in advanced lithium-ion batteries. Adv. Funct. Mater. **28**, 1704858 (2018). https://doi.org/10.1002/adfm.201704858

[S12] Y. Zhang, L. Wang, H. Xu, J. Cao, D. Chen et al., 3D chemical cross-linking structure of black phosphorus@CNTs hybrid as a promising anode material for lithium-ion batteries. Adv. Funct. Mater. **30**, 1909372 (2020). https://doi.org/10.1002/adfm.201909372

[S13] G. Li, J.Y. Li, F.S. Yue, Q. Xu, T.T. Zuo et al., Reducing the volume deformation of high capacity SiO_x/G/C anode toward industrial application in high energy density lithium-ion batteries. Nano Energy **60**, 485-492 (2019). https://doi.org/10.1016/j.nanoen.2019.03.077

[S14] R.J.C. Dubey, P.V.W. Sasikumar, N. Cerboni, M. Aebli, F. Krumeich et al., Silicon oxycarbide-antimony nanocomposites for high-performance Li-ion battery anodes. Nanoscale **12**, 13540–13547 (2020). https://doi.org/10.1039/D0NR02930K

[S15] T. Meng, B. Li, Q. Wang, J. Hao, B. Huang et al., Large-scale electric-field confined silicon with optimized charge-transfer kinetics and structural stability for high-rate lithium-ion batteries. ACS Nano **14**, 7066-7076 (2020). https://doi.org/10.1021/acsnano.0c01796

[S16] P. Nie, Z. Le, G. Chen, D. Liu, X. Liu et al., Graphene caging silicon particles for high-performance lithium-ion batteries. Small **14**, 1800635 (2018). https://doi.org/10.1002/smll.201800635

[S17] N. Ding, J. Xu, Y.X. Yao, G. Wegner, X. Fang et al., Determination of the diffusion coefficient of lithium ions in nano-Si. Solid State Ion. **180**, 222-225 (2009). https://doi.org/10.1016/j.ssi.2008.12.015

[S18] Y. Xiang, L. Xu, L. Yang, Y. Ye, Z. Ge et al., Natural stibnite for lithium-/sodium-ion batteries: Carbon dots evoked high initial coulombic efficiency. Nano-Micro Lett. **14**, 136 (2022). https://doi.org/10.1007/s40820-022-00873-x

[S19] Y. Tang, Y. Wei, A.F. Hollenkamp, M. Musameh, A. Seeber et al., Electrolyte/structure-dependent cocktail mediation enabling high-rate/low-plateau metal sulfide anodes for sodium storage. Nano-Micro Lett. **13**, 178 (2021). https://doi.org/10.1007/s40820-021-00686-4
